# Oral administration of live combined *Bacillus subtilis* and *Enterococcus faecium* alleviates colonic oxidative stress and inflammation in osteoarthritic rats by improving fecal microbiome metabolism and enhancing the colonic barrier

**DOI:** 10.3389/fmicb.2022.1005842

**Published:** 2022-11-10

**Authors:** Jilang Tang, Xiaopeng Song, Mingchao Zhao, Hong Chen, Yingying Wang, Binger Zhao, Shiming Yu, Tianwen Ma, Li Gao

**Affiliations:** ^1^Heilongjiang Key Laboratory for Laboratory Animals and Comparative Medicine, Harbin, China; ^2^College of Veterinary Medicine, Northeast Agricultural University, Harbin, China

**Keywords:** osteoarthritic, *Bacillus subtilis* and *Enterococcus faecium*, inflammatory response, oxidative stress, fecal flora, untarget metabolism, colonic barrier

## Abstract

Osteoarthritis (OA) causes intestinal damage. The protective effect of probiotics on the intestine is indeed effective; however, the mechanism of protection against intestinal damage in OA is not clear. In this study, we used meniscal/ligamentous injury (MLI) to mimic OA in rats and explored the colonic protective effects of *Bacillus subtilis* and *Enterococcus faecium* on OA. Our study showed that treatment with *B. subtilis* and *E. faecium* attenuated colonic injury and reduced inflammatory and oxidative stress factors in the serum of osteoarthritic rats. α- and ß diversity of the fecal flora were not different among groups; no significant differences were observed in the abundances of taxa at the phylum and genus levels. We observed the presence of the depression-related genera *Alistipes* and *Paraprevotella*. Analysis of fecal untargeted metabolism revealed that histamine level was significantly reduced in the colon of OA rats, affecting intestinal function. Compared to that in the control group, the enriched metabolic pathways in the OA group were primarily for energy metabolisms, such as pantothenate and CoA biosynthesis, and beta-alanine metabolism. The treatment group had enriched linoleic acid metabolism, fatty acid biosynthesis, and primary bile acid biosynthesis, which were different from those in the control group. The differences in the metabolic pathways between the treatment and OA groups were more evident, primarily in symptom-related metabolic pathways such as Huntington's disease, spinocerebellar ataxia, energy-related central carbon metabolism in cancer, pantothenate and CoA biosynthesis metabolic pathways, as well as some neurotransmission and amino acid transport, and uptake- and synthesis-related metabolic pathways. On further investigation, we found that *B. subtilis* and *E. faecium* treatment enhanced the colonic barrier of OA rats, with elevated expressions of tight junction proteins occludin and Zonula occludens 1 and MUC2 mRNA. Intestinal permeability was reduced, and serum LPS levels were downregulated in the treatment group. *B. subtilis* and *E. faecium* also regulated the oxidative stress pathway Keap1/Nrf2, promoted the expression of the downstream protective proteins HO-1 and Gpx4, and reduced intestinal apoptosis. Hence, *B. subtilis* and *E. faecium* alleviate colonic oxidative stress and inflammation in OA rats by improving fecal metabolism and enhancing the colonic barrier.

## Introduction

Osteoarthritis (OA) is a chronic inflammatory disease of the joints involving the cartilage and its surrounding tissues and is manifested by joint pain and dysfunction caused by substantial joint degeneration (Robinson et al., 2016). It is the main contributor to global disability (Cross et al., 2014), and OA in the knee area is the most prevalent. OA has been reported as a metabolic disease (Zhuo et al., 2012), and it can coexist with obesity and diabetes (Wang and He, 2018; Veronese et al., 2019). Obesity and OA have similar manifestations, including chronic low-grade inflammation. Furthermore, an association between type 2 diabetes and OA has been established; however, a causal relationship remains to be elucidated. Type 2 diabetes plays a role in OA primarily through chronic hyperglycemia-induced oxidative stress in the joint tissue, insulin resistance (Veronese et al., 2019), and leptin-associated chondrocyte aging (Zhao et al., 2019). Inflammation and oxidative stress are commonly observed in OA and have become therapeutic targets (Liu-Bryan, 2015; Wang et al., 2018).

Probiotics are living microorganisms that confer health benefits to the host when administered in adequate amounts. Due to their safety, probiotics are increasingly regarded as food rather than medicine (de Simone, 2019). It can balance the microbial ecosystem in the intestinal tract, and some metabolites of probiotics have antibacterial and anti-inflammatory activities, which can improve intestinal damage caused by pathogenic microorganisms (Aw and Fukuda, 2019). Probiotics have shown significant applications in some intestinal diseases, such as ulcerative colitis, irritable bowel syndrome, and Crohn's disease, and can improve dysregulated flora (Kim et al., 2019). *Bacillus subtilis* is a highly adaptable bacterium that can grow in a variety of environments, including soil, plant roots, and the gastrointestinal tract of animals. Furthermore, *B. subtilis* can induce heat shock proteins to protect intestinal cells from oxidative tissue damage and loss of barrier function (Williams, 2007). Metagenomics has revealed that ~4% of the *B. subtilis* genome produces secondary metabolites, some of which have anti-fungal and antibacterial functions (Earl et al., 2008). In contrast, *Enterococcus faecium* is safe when used in dairy fermentation (Ogier and Serror, 2008), and it can promote the growth performance of weaning piglets, protect the morphology of the intestinal villi, improve immunity, and modulate the intestinal flora (Wang et al., 2019). *B. subtilis* can suppress oxidative stress in the intestine of laying hens by modulating the intestinal flora and related metabolites (Zou et al., 2022). *E. faecium* reduces reactive oxygen species (ROS) in the epithelial cells of pigs infected with *Salmonella typhimurium* and *Escherichia coli* (Palkovicsne Pezsa et al., 2022). Therefore, both *B. subtilis* and *E. faecium* have effective anti-inflammatory and antioxidant properties and are usually used in combination to enhance their probiotic effect (Pi et al., 2022).

OA is related to intestinal health, and many reports have confirmed that OA is related to changes in the intestinal microbial ecosystem. Chronic systemic inflammation and changes in the intestinal flora of OA can be triggered and accelerated by obesity. Activation of inflammation in obesity is also caused by changes in the intestinal microbiota; therefore, the intestinal flora has been used as a target for the treatment of obesity-induced OA (Schott et al., 2018; Liu et al., 2019). Fecal transplantation from individuals with metabolic OA to germ-free mice resulted in increased blood levels of LPS, decreased mRNA expression of the tight junction protein Zonula occludens 1 (ZO-1), and increased intestinal permeability in germ-free mice (Huang et al., 2020). Simultaneously, OA was prevented through gut health interventions, which in turn promoted joint repair through the gut-muscle-joint axis (Xu et al., 2022).

The treatment and improvement of osteoarthritis involve bone tissue. The growth and recovery of bone cells depend on the intestinal tract to absorb minerals and vitamins. In addition, there is a close relationship between bones and the immune system, with various cytokines secreted by immune cells affecting bone development (Bhardwaj et al., 2022). The contribution of probiotics to immunity has been demonstrated (Klaenhammer et al., 2012). It is reasonable to believe that probiotics have an impact on bone. Probiotics have been shown to improve bone disease. Supplementation with *B.subtilis* reduced bone loss due to periodontitis in rats. *Lactobacillus* and *Bifidobacterium* have been shown to prevent ovariectomy-mediated bone loss (Sojan et al., 2022). Although the effects of probiotics on osteoarthritis are less well reported at present, there is a growing amount of evidence emerging. Some *Lactobacillus plantarum* strains may mitigate the development of osteoarthritis by reducing inflammation and pain, improving subchondral bone structure, and protecting against ACLT-induced cartilage damage (I et al., 2022). Probiotic supplementation can prevent the growth of various pathogenic bacteria, thereby restoring the composition of the intestinal gut flora and preventing dysbiosis-mediated bone loss. Probiotics can also directly modulate bone cells, such as *Bifidobacterium longum* and *Lactobacillus reuteri*, and can enhance bone formation by decreasing intestinal permeability and increasing the absorption of calcium and vitamins (Bhardwaj et al., 2022). Oral inactivation of *Bifidobacterium longum* cultures reduced structural cartilage lesions and decreased type II collagen degradation in OA guinea pigs (Henrotin et al., 2021). Oral administration of *C. butyricum* slowed or even reversed ACLT-induced knee OA progression in rats, which may be related to butyrate enhancing the intestinal barrier and reducing systemic inflammation (Chang et al., 2022). The use of *B. subtilis* and *E. faecium* in OA has not been reported, but *B. subtilis* has an effect on bone loss, and *B. subtilis* and *E. faecium* have anti-inflammatory and antioxidant properties. Therefore, we hypothesize that the combination of these two bacteria can improve the intestinal status of OA animals and even attenuate the effects of osteoarthritis.

Since OA is a type of systemic low-grade chronic inflammation, the occurrence of inflammation and the production of inflammatory factors are closely related to the host's metabolism. Metabolism in the joint tissue and blood has been used to explain the occurrence and development of OA and to determine diagnostic biomarkers. However, studies on the intestinal metabolism of patients with OA or infected animals are relatively rare. This study focused on the changes in the fecal flora and metabolites in osteoarthritic rats to investigate the effects of oral administration of combined *B. subtilis* and *E. faecium* on the colonic environment and explore the possible mechanisms of probiotics in improving the colonic health of osteoarthritic rats.

## Materials and methods

### Bacterial strains

Live combined *B. subtilis* and *E. faecium* were produced by Beijing Hanmei Pharmaceutical Co., Ltd. (Beijing, China, approval number: S20030087). Each 250 mg sample contained live *B. subtilis* (R-179 5.0 × 10^7^) and *E. faecium* R-026 (4.5 × 10^8^).

### Experimental animals and sample collection

Fifteen SD male eight-week-old rats were purchased from Changchun Yisi Experimental Animal Technology Co., Ltd. (Changchun, China, certificate number: 2020000034399, license number: SCXK [JI]-2018-0007) and used in the experiment. The rats were divided into three treatment groups (*n* = 5): the control group (CON), the OA group, and the OA treated with *B. subtilis* and *E. faecium* group (TREAT group). All rats were reared in the same environment for 1 week after purchase and were given formulated commercial rat chow. The rates were fed diet and sterile water ad libitum. One week later, two groups underwent meniscal/ligament injury (MLI) surgery to establish artificial OA models. The CON and OA groups were orally administered 2 ml of sterile saline solution daily, and the TREAT group was administered 250 mg of the live combined bacteria dissolved in 2 ml of sterile saline solution daily. All treatments were administered for 30 days. Then, all rats were euthanized after 30 d. Serum was collected from each rat, and fresh colonic tissue was acquired and placed in sterile freezing tubes and frozen immediately with liquid nitrogen. Fresh feces (0.5 g) from three rats in each group were collected. Two portions of the fecal sample were collected from each rat separately. The samples were stored in sterile ultra-low temperature storage tubes and frozen with liquid nitrogen. The intestinal flora and metabolites were examined separately.

### Serum inflammatory markers, oxidative stress levels, and intestinal function evaluation

Blood was collected from the tail vein and then centrifuged at 5,000 rpm for 15 min. Interleukin-1ß (IL-1ß), Interleukin-6 (IL-6), malondialdehyde (MDA), glutathione (GSH), superoxide dismutase (SOD), inducible Nitric Oxide Synthase (iNOS), Diamine oxidase (DAO), C-reactive protein (CRP), D-lactate (D-LAC), and LPS in serum were determined using ELISA kits (Jiangsu Jingmei Biocompany, Yancheng, China), according to the manufacturer's instructions.

### Detection of colonic mucin MUC2

The total RNA of each colon sample was extracted using the RNA Simple Total RNA Kit (Tiangen Biotech Co., Ltd., Beijing, China). Complementary DNA (cDNA) was synthesized from 2 μg of total RNA *via* reverse transcription using the TransScript^®^ All-in-One First-Strand cDNA Synthesis SuperMix for PCR (Transgen Biotech Co., Ltd., Beijing, China), according to the manufacturer's instructions. Amplification (40 cycles) was conducted using the SYBR Green SuperReal PreMix Color, forward and reverse primers of MUC2 (Table 1), cDNA, and ddH_2_O. The LightCycler^®^480 (Roche, Germany) was used to monitor the fluorescence intensity during the entire PCR process to obtain the cycle threshold (Ct).

**Table 1:** Primer sequence.

**Target gene**	**Forward**	**Reverse**
MUC2	ACCACCATTACCACCACCTCAG	CGATCACCACCATTGCCACTG

### Histological analysis

Colon samples were fixed with 4% paraformaldehyde solution, embedded in paraffin, and cut into sections (4 μm). Subsequently, the samples were stained with hematoxylin and eosin and observed under light microscopy at 40 × magnification.

### Immunohistochemistry

Paraffin sections were dewaxed in xylene and rehydrated in decreasing concentrations of ethanol. The sections were permeabilized with 0.5% Triton X-100 for 15 min, followed by washing three times with HBSS. Subsequently, the samples were submerged in 3% H_2_O_2_ to remove catalase. For ZO-1 staining, sections were incubated with anti-ZO-1 rabbit pAb (1:200, ABclonal, USA) overnight at 4°C in a humidified box. For occludin staining, sections were incubated with anti-occludin rabbit pAb (1:100, Wanlei, China) overnight at 4°C. The sections were then incubated with goat anti-rabbit antibody (1:200, Boster, China) for 1 h at 37°C and washed.

### Total fecal DNA extraction

Total genomic DNA was extracted from each sample using the OMEGA Soil DNA Kit (M5635-02) (Omega Bio-Tek, Norcross, GA, USA), then stored at −20°C before analysis. The quantity and quality of the extracted DNA were measured using a NanoDrop NC2000 spectrophotometer (Thermo Fisher Scientific, Waltham, MA, USA) and agarose gel electrophoresis, respectively.

### 16S rRNA gene amplicon sequencing

The V3–V4 region of the bacterial 16S rRNA gene was amplified using the forward primer 338F (5′-ACTCCTACGGGAGGCAGCA-3′) and reverse primer 806R (5′-GGACTACHVGGGTWTCTAAT-3′). Sample-specific 7-bp barcodes were incorporated into the primers for multiplex sequencing. The PCR components contained 5 μl of 5 × buffer, 0.25 μl of Fast pfu DNA Polymerase (5 U/μl), 2 μl of 2.5 mM dNTPs, 1 μl of 10 μM forward and reverse primers, 1 μl of the DNA template, and 14.75 μl of ddH_2_O. Thermal cycling consisted of initial denaturation at 98°C for 5 min; followed by 25 cycles of denaturation at 98°C for 30 s, annealing at 53°C for 30 s, and extension at 72°C for 45 s; and a final extension of 5 min at 72°C. PCR amplicons were purified using the Vazyme VAHTSTM DNA Clean Beads (Vazyme, Nanjing, China) and quantified using the Quant-iT PicoGreen dsDNA Assay Kit (Invitrogen, Carlsbad, CA, USA). After the individual quantification step, amplicons were pooled in equal amounts, and pair-end (2×250 bp) sequencing was performed using the Illumina MiSeq platform with MiSeq Reagent Kit v3 at Shanghai Personal Biotechnology Co., Ltd. (Shanghai, China).

### Bioinformatics

Microbiome bioinformatics was performed using QIIME2 2019.4, with slight modifications from the official tutorial^1^ Briefly, raw sequence data were demultiplexed using the demux plugin, followed by primer cutting using the cutadapt plugin. Subsequently, sequences were quality filtered, denoised, and merged, and the chimera was removed using the DADA2 plugin. Non-singleton amplicon sequence variants (ASVs) were aligned using MAFFT. Sequence data analyses were primarily performed using QIIME2 and R packages (v3.2.0). ASV-level alpha diversity indices, such as the Chao1 richness index, Shannon diversity index, and Simpson index, were calculated using the ASV table in QIIME2 and visualized as box plots. Beta diversity analysis was performed to investigate the structural variation of microbial communities across samples using Bray-Curtis metrics and visualized *via* the unweighted pair-group method with arithmetic means hierarchical clustering. Venn diagram was generated to visualize the shared and unique ASVs among samples or groups using the R package “VennDiagram,” based on the occurrence of ASVs across samples/groups regardless of their relative abundance. Taxonomic composition and abundance were visualized using MEGAN and GraPhlAn. Linear discriminant analysis effect size (LEfSe) was performed to detect differentially abundant taxa across groups using the default parameters.

### Liquid chromatography (LC) tandem mass spectrometry (MS) analysis

The fecal samples were thawed at 4°C, and then an appropriate amount was added with cooled methanol/acetonitrile/water solution (2:2:1, v/v). Then, the resulting mixture was mixed using a vortex, subjected to low-temperature ultrasound for 30 min at −20°C, incubated for 10 min, and then centrifuged at 14,000 × *g* for 20 min. The supernatant was collected and vacuum-dried. For MS, 100 μL of acetonitrile aqueous solution (acetonitrile:water =1:1, v/v) was added for resolution and then centrifuged for 15 min at 14,000 × *g*, 4°C. The supernatant was collected and used for analysis.

Analyses were performed using a UHPLC (1290 Infinity LC, Agilent Technologies) coupled to a quadrupole time-of-flight (AB Sciex TripleTOF 6600) at Shanghai Applied Protein Technology Co., Ltd.

For HILIC, samples were analyzed using the 2.1 mm × 100 mm ACQUIY UPLC BEH 1.7 μm column (Waters, Ireland). In both the ESI positive and negative modes, the mobile phase contained 25 mM ammonium acetate and 25 mM ammonium hydroxide in water (A), and acetonitrile (B). The gradient was set as follows: 85% B for 1 min, 65% B for 11 min, 40% B for 0.1 min maintained for 4 min, and finally 85% B in 0.1 min, with a 5 min re-equilibration period.

For RPLC, the 2.1 mm × 100 mm ACQUIY UPLC HSS T3 1.8 μm column (waters, Ireland) was used. In the ESI positive mode, the mobile phase contained water with 0.1% formic acid (A) and acetonitrile with 0.1% formic acid (B); in the ESI negative mode, the mobile phase contained 0.5 mM ammonium fluoride in water (A) and acetonitrile (B). The gradient was set as follows: 1% B for 1.5 min, 99% B for 11.5 min, kept for 3.5 min, and finally 1% B for 0.1 min, with a 3.4 min re-equilibration period. The flow rate was 0.3 mL/min, and the column temperature was kept constant at 25°C. A 2 μL aliquot of each sample was injected. The ESI source conditions were set as follows: ion source gas 1 (Gas1) at 60, ion source gas 2 (Gas2) at 60, curtain gas (CUR) at 30, source temperature: 600°C, and ion spray voltage floating at ± 5,500 V. For MS, the m/z was set at 60–1,000 Da, and the accumulation time for TOF-MS scan was set at 0.20 s/spectrum. For the auto MS/MS, the m/z was set at 25–1,000 Da, and the accumulation time for the product ion scan was set at 0.05 s/spectrum. The product ion scan was acquired using information-dependent acquisition with a high sensitivity mode, and the parameters were set as follows: collision energy (CE), 35 V with ± 15 eV; declustering potential (DP), 60 V (+) and −60 V (–); excluding isotopes, within 4 Da; and candidate ions to monitor per cycle: 10.

The raw MS data (wiff.scan files) were converted to MzXML files using ProteoWizard MSConvert before being imported into the XCMS software. For peak picking, the following parameters were used: centWave *m*/*z* = 25 ppm, peak width = *c* (10, 60), and prefilter = *c* (10, 100). For peak grouping, the following parameters were used: bw = 5, mzwid = 0.025, minfrac = 0.5. The Collection of Algorithms of MEtabolite pRofile Annotation was used for the annotation of isotopes and adducts. In the extracted ion features, only the variables with more than 50% of the nonzero measurement values in at least one group were kept. Compound identification of metabolites was performed by comparing the m/z value (<25 ppm), and MS/MS spectra with an in-house database established with available authentic standards.

After normalization to the total peak intensity, the processed data were subjected to multivariate data analysis, including Pareto-scaled principal component analysis (PCA), partial least-squares discrimination analysis (PLS-DA), and orthogonal partial least-squares discriminant analysis (OPLS-DA), using the R package ropls. The seven-fold cross-validation and response permutation testing were performed to evaluate the robustness of the model. Using univariate analysis, the difference among all metabolites (including unidentified metabolites) detected in positive and negative ion modes was analyzed. KEGG pathway analysis was also performed. A OPLS-DA VIP > 1 and *P*-value < 0.05 indicated significant differences.

### Western blot

The total protein of each colon sample was extracted using RIPA lysis buffer, and the concentration and purity were measured using a BCA kit. SDS-PAGE and PVDF membrane were used to separate and transfer the proteins. The membranes were blocked with 5% skimmed milk powder for 2 h and incubated overnight at 4°C with the primary antibodies against Bax (1:1,000, Wanlei, China), Nrf2 (1:1,000, Wanlei), Gpx4 (1:5,000, Abcam, Britain), Keap 1(1:1,000, Wanlei), Ho-1 (1:2000, ABclonal, USA), and ß-actin (1:500, Wanlei). Subsequently, the membranes were washed three times with Tris-buffered saline Tween-20 (TBST) for 10 min and incubated with the secondary antibody (1:3,000, Zhongshan Jinqiao, China) at 25°C for 1 h. Membranes were washed three times with TBST, and protein signals were developed using an ECL kit. Images were captured using a gel imaging system and quantitatively analyzed using the ImageJ software. The experiment was performed in triplicate.

### Immunofluorescence

After dehydration and rehydration, colonic slides were incubated with 0.1% trypsin-EDTA solution at room temperature for 10 min to remove the antigen. The slides were then blocked with 10% normal goat serum for 1 h at 25°C. Samples were incubated with Anti-Nrf2 rabbit pAb (1:100 dilution, Wanlei) at 4°C for 12 h. Subsequently, the secondary antibodies, including PE-conjugated goat anti-rabbit IgG (1:200 dilution, Zhongshan Jinqiao) and conjugated goat anti-rabbit IgG (1:200, Zhongshan Jinqiao) were incubated for 30 min. Images were obtained using the LeicaDMI3000 + DFC310FX fluorescence microscope (Leica, Germany). Three images were selected from each group for quantitative analysis using ImageJ.

### Statistical analysis

GraphPad Prism 8.0 (GraphPad Software, Inc., San Diego, CA, USA) was used to analyze the data. Data are expressed as the mean ± SD. A one-way analysis of variance between multiple groups was performed, and a *p* < 0.05 was considered statistically significant.

## Results

### *B. subtilis* and *E. faecium* can reduce colonic histopathological injury

**Figure 1:**
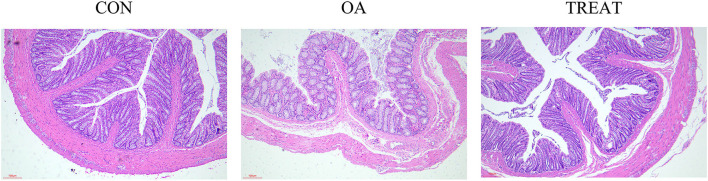
Effects of the oral administration of live combined *Bacillus subtilis* and *Enterococcus faecium* on the histopathological changes in OA-induced rat colon. H&E staining images of each group (*n* = 5). Representative photomicrographs are at 200 × magnification.

Colonic H&E staining showed the ameliorative effect of *B. subtilis* and *E. faecium* on the colonic injury. The villi and crypts of the colon were tightly and neatly arranged in the CON and TREAT groups; in the OA group, the colonic villi were incomplete, and the epithelial space became wider than that in the CON or TREAT group. Furthermore, we found that OA led to colonic lamina propria infiltration with inflammatory cells, and venous congestion was observed in the lamina propria, while treatment reversed the colonic injury (Figure 1).

### *B. subtilis* and *E. faecium* resist inflammation, reduce oxidative stress, and maintain intestinal function

**Figure 2:**
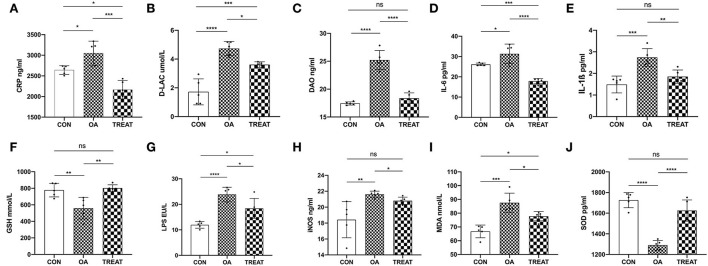
Effects of the oral administration of live combined *Bacillus subtilis* and *Enterococcus faecium* on serum intestinal function parameters, antioxidant parameters, and inflammatory cytokine and LPS levels in OA-induced rats with colitis. **(A)** CRP. **(B)** D-LAC. **(C)** DAO. **(D)** IL-6. **(E)** IL-1ß. **(F)** GSH. **(G)** LPS. **(H)** iNOS. **(I)** MDA. **(J)** SOD. Data are presented as the mean ± SD; *n* = 5, *p < 0.05, **p < 0.01, ***p < 0.001 and ****p < 0.0001.

Compared to the CON group, the levels of serum CRP, D-LAC, DAO, IL-6, and IL-1ß were significantly increased in the OA group and were significantly decreased after 30 d treatment with *B. subtilis* and *E. faecium* (Figures 2A–E). Serum LPS and increased intestinal permeability were observed in the OA group and were significantly decreased in the TREAT group (Figure 2G). This indicated that colonic permeability decreased in the TREAT group. Furthermore, iNOS and MDA were significantly increased in the OA group compared to the CON group, while these factors were decreased in the TREAT group (Figures 2H,I). Serum GSH and SOD levels were significantly decreased in the OA group and were effectively restored in the TREAT group (Figures 2F,J).

### Analysis of the fecal flora among groups

**Figure 3:**
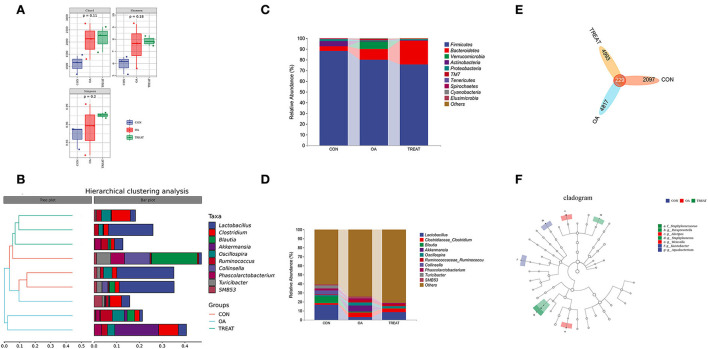
Effects of the oral administration of live combined *Bacillus subtilis* and *Enterococcus faecium* on the fecal microbiota composition of OA rats. **(A)** α diversity. **(B)** Hierarchical clustering analysis. **(C)** Community bar plots of phylum level. **(D)** Community bar plots of the genus level. **(E)** Venn diagram demonstrates the overlaps among groups. **(F)** Taxonomic cladogram of LEfSe analysis (*n* = 3).

To better understand the changes in the colonic environment of osteoarthritic rats and the effects of *B. subtilis* and *E. faecium* on the colonic tract, we examined the fecal flora of rats. Each experimental group had three parallel samples for the detection of fecal bacteria 16S rRNA. A total of 830,303 sequences were detected in all samples. After processing the sequences using DADA2, 480,187 high-quality chimera-removing sequences were obtained, and the non-singleton sequences were 472,464. Sample sequence numbers are shown in Supplementary Table 1. For the taxonomic classification, 58 phyla, 45 classes, 4,574 orders, 7,241 families, and 2,575 genera were annotated (Supplementary Table 2). Diversity analysis at the phylum and genus levels revealed no difference in the α-diversity indices (Chao1, Shannon, and Simpson) of the fecal flora among the groups (Figure 3A). Meanwhile, the β-diversity analysis showed that the top 10 abundant genera in each group were closely clustered in the hierarchy (Figure 3B). In addition, no difference was observed in the relative abundance of the top 10 phyla and genera (Figures 3C,D). Among the dominant phyla, *Bacteroidetes* was abundant in the OA and TREAT groups, while *Firmicutes* was abundant in the CON group. At the genus level, *Lactobacillus* was in abundance in the CON and TREAT groups, while *Clostridiaceae_Clostridium, Akkermansia, Oscillospira, Ruminococcaceae*, and *Ruminococcus* were more abundant in the OA group. Figure 3E shows the number of ASVs in each group: CON group, 2097; OA group, 4817; and TREAT group, 4993; the three groups shared 229 ASVs. To further understand the specific predominant bacteria in the three groups, LEfSe was used to compare microbial composition (Figure 3F). Ultimately, seven taxa (LDA > 2, *p* < 0.05) were identified as significantly decreased, among which *Aquabacterium* and *Kaistobacter* were the differential species in the CON group, *Weissella* and *Alistipes* in the OA group, and *Paraprevotella, Staphylococcaceae*, and *Staphylococcus* in the TREAT group. *Alistipes* and *Paraprevotella* were associated with depression, which is a novel finding in the study.

### Non-targeted metabolic analysis

To further understand the changes in the intestinal environment, we performed a non-targeted metabolic analysis using fecal samples. A total of 424 metabolites were identified, including 261 positive ions and 163 negative ions (Supplementary Table 3).

**Figure 4:**
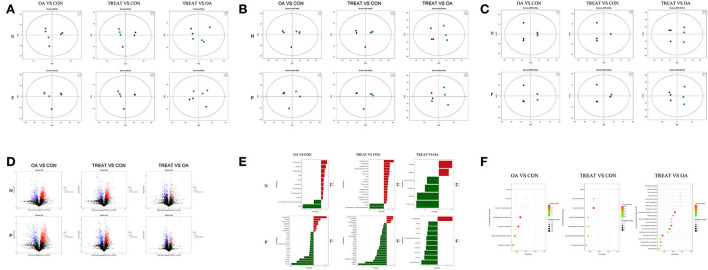
Effects of the oral administration of live combined *Bacillus subtilis* and *Enterococcus faecium* on the metabolite analysis of the feces of OA rats. **(A)** Score plot of PCA. **(B)** Score plot of PLS-DA. **(C)** Score plot of OPLS-DA. **(D)** Volcano plot shows the number of dysregulated metabolites. **(E)** Differential metabolites. **(F)** Differential metabolic pathway (*n* = 3).

The principal component analysis showed that the positive and negative ions between the groups had a clearer separation trend (Figure 4A), indicating differences between the groups. The PLS-DA (Figure 4B) and OPLS-DA (Figure 4C) model score graphs showed that the samples in different groups were completely separated, indicating that the model can effectively distinguish the samples in different groups.

Based on univariate analysis, a differential analysis of all metabolites (including unidentified metabolites) detected in the positive and negative ion modes was performed. Different metabolites with FC> 1.5, FC <0.67, and *P*-value <0.05 were visualized using a volcano graph (Figure 4D).

Non-targeted metabolomics usually uses strict OPLS-DA VIP>1 and *P*-value <0.05 as the screening criteria to differentiate metabolites, which was applied in the present study. The significantly different metabolites are shown in Figure 4E. The expressions of metabolites upregulated by the negative ions in the OA group compared with those in the CON group were pentadecanoic acid, pantothenate, docosatrienoic acid, uracil, nicotinate, 4-pyridoxic acid, cis-9-palmitoleic acid, 3-phenylpropanoic acid, and L-arabinose. The metabolites downregulated by the negative ions were 1,3,5(10)-estratrien-3,17. beta-diol 17-glucosiduronate and taurine. Meanwhile, the metabolites upregulated by the positive ion mode were phenylethylamine, resorcinol, N6-acetyl-L-lysine, hypoxanthine, pyrrolidine, sphinganine, and 7-oxocholesterol. The downregulated metabolites were Ser-Leu, Thr-Arg, Ala-Lys, Val-Arg, Gly-Arg, S-methyl-5′-thioadenosine, Ile-Arg, Val-Ile, Pro-Arg, Ala-Leu, Val-Lys, His-Ile, histamine, creatinine, and glycitein.

Compared to the CON group, the TREAT group had upregulated pentadecanoic acid, phenylpyruvate, docosatrienoic acid, tridecanoic acid (tridecylic acid), capric acid, myristic acid, uracil, xanthine, azelaic acid, 4-pyridoxic acid, nicotinate, 2E-eicosenoic acid, cis-9-palmitoleic acid, 13-OxoODE, 3-phenylpropanoic acid, chenodeoxycholate, and palmitic acid; 1,3,5(10)-estratrien-3,17. beta-diol 17-glucosiduronate and taurine were downregulated. In contrast, tetrahydro-l-biopterin, PC(16:0/16:0), pyrrolidine, and N6-Methyladenine were upregulated, while Ile-Pro, 5-methylcytosine, Ile-Glu, Pro-Arg, Ser-Leu, L-methionine, Gly-Arg, 2-hydroxyadenine, Gly-Ile, Ile-Ile, Val-Arg, Lys-Thr, Ala-Lys, Val-Ile, Ile-Arg, Ala-Leu, Ile-Ala, creatinine, Thr-Arg, Val-Lys, S-methyl-5′-thioadenosine, His-Ile, histamine, and glycitein were downregulated.

Compared to the OA group, the fecal metabolites that were upregulated by negative ions in the TREAT group were taurocholate and palmitic acid, and those that were downregulated were N-acetyl-DL-methionine, L-phenylalanine, pantothenate, and acetyl-DL-leucine. The only metabolite upregulated in the positive ion mode was cyclohexylamine, while the downregulated metabolites were Leu-Phe, Phe-Phe, L-valine, L-aminocyclopropanecarboxylic acid, L-citrulline, L-glutamate, L-methionine, D-proline, and N-oleoylethanolamine.

The screened differential metabolites were annotated and analyzed using the KEGG database, and the metabolic differences between the three groups were further analyzed using KEGG pathway enrichment analysis (Figure 4F). Compared to the CON group, the fecal metabolism in the OA group was enriched in pathways involving asthma, pertussis, Fc ε RI signaling pathway, pantothenate and CoA biosynthesis, ß-alanine metabolism, neuroactive ligand-receptor interaction, and phenylalanine metabolism. Compared to the CON group, the fecal metabolism of the TREAT group was enriched in pathways involving asthma, linoleic acid metabolism, fatty acid biosynthesis, primary bile acid biosynthesis, protein digestion and absorption, neuroactive ligand-receptor interaction, and phenylalanine metabolism.

Compared to the OA group, the fecal metabolism of the TREAT group was enriched FoxO signaling pathway, Huntington's disease, nicotine addiction, cocaine addiction, spinocerebellar ataxia, long-term potentiation, glutamatergic synapse, circadian entrainment, central carbon metabolism in cancer, mineral absorption, taurine and hypotaurine metabolism, arginine biosynthesis, protein digestion and absorption, aminoacyl-tRNA biosynthesis, pantothenate and CoA biosynthesis, biosynthesis of amino acids, cysteine and methionine metabolism, 2-oxocarboxylic acid metabolism, arginine and proline metabolism, and ABC transporters.

### *B. subtilis* and *E. faecium* treatment enhanced the colonic tissue barrier

**Figure 5:**
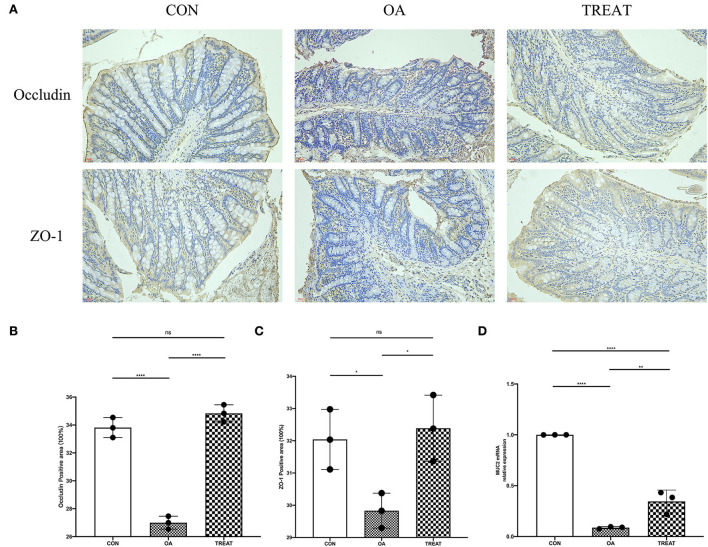
Effect of the oral administration of live combined *Bacillus subtilis* and *Enterococcus faecium* on OA colonic tight junction protein and mucin mRNA expression. Representative photomicrographs are at 400 × magnification. **(A)** Immunohistochemical staining of occludin and ZO-1. **(B)** Positive-stained area for occludin. **(C)** Positive-stained area for ZO-1. **(D)** MUC2 mRNA relative expression. All data are expressed as the mean± SD. *p < 0.05, **p < 0.01 and ****p < 0.0001; *n* = 3.

Results showed that probiotic treatment significantly improved the clinical performance of rats with OA. Based on previous studies in our laboratory, we suspected that these improvements were achieved by probiotics by enhancing the barrier function of the colon. Colonic immunohistochemical analysis showed that the expression of colonic barrier proteins occludin and ZO-1 were significantly reduced in the OA group, whereas the expression of these proteins could be significantly increased by the *B. subtilis* and *E. faecium* treatment (Figure 5A). We also explored the mRNA expression of MUC2, which was significantly lower in the OA group than in the CON group and was significantly higher in the TREAT group than in the OA group (Figure 5B).

### *B. subtilis* and *E. faecium* treatment alleviated oxidative stress and reduced apoptosis in the colon of osteoarthritic rats

**Figure 6:**
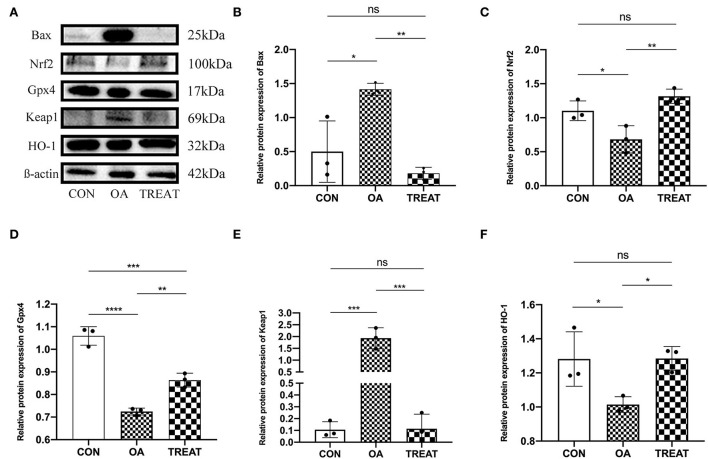
Effect of the oral administration of live combined *Bacillus subtilis* and *Enterococcus faecium* on the OA colonic protein expression of Bax, Nrf2, Gpx4, Keap1, and HO-1. **(A)** Protein expressions detected *via* western blot. **(B–F)**. Protein expression analysis of Bax, Nrf2, Gpx4, Keap1, and HO-1. All data are expressed as the mean± SD. *p < 0.05, **p < 0.01, ***p < 0.001 and ****p < 0.0001; *n* = 3.

We then explored the protein expression of the oxidative stress pathway Keap1/Nrf2 in colonic tissues using western blot. Results showed that the relative expression of Keap1 protein was significantly higher in the OA group than in the CON group, while the expression of Nrf2 was decreased. Opposite results were observed in the TREAT group (Figures 6A,C,E). We also examined the oxidative stress-related protective proteins HO-1 and Gpx4 and found that the expression levels of HO-1 and Gpx4 were decreased in the OA group and could be restored after treatment with *B. subtilis* and *E. faecium* (Figures 6A,D,F). We also examined the expression of Bax, an apoptosis-related protein in the colon, and found an increased expression in the OA group, whereas treatment with *B. subtilis* and *E. faecium* significantly reduced the relative expression of Bax (Figures 6A,B).

### *B. subtilis* and *E. faecium* treatment can effectively enhance Nrf2 protein expression and promote its entry into the nucleus

**Figure 7:**
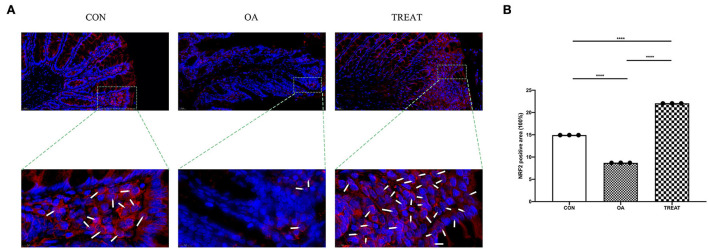
Effect of the oral administration of live combined *Bacillus subtilis* and *Enterococcus faecium* on the OA colonic protein expression of Nrf2 measured *via* immunofluorescence. Representative photomicrographs are at 400 × magnification. The magnified parts are 1,600 × magnification. **(A)** Immunofluorescence staining. White arrows indicate Nrf2 entering the nucleus. **(B)** Positive-stained are for Nrf2. Data are expressed as the mean± SD. ****p < 0.001. *n* = 3.

We found that *B. subtilis* and *E. faecium* could regulate the oxidative stress pathway in the colon tissue of osteoarthritic rats using western blot. To further understand the mechanism underlying oxidative stress alleviation, we performed immunofluorescence probing for Nrf2. We found that the fluorescence intensity of Nrf2 in the colonic tissues was enhanced after treatment with *B. subtilis* and *E. faecium*, which was consistent with the results of the western blot. Also, we observed the localization of Nrf2 expression *via* immunofluorescence and found that the expression of Nrf2 was not only increased in the colon tissue, but also in the nucleus of colonic villous epithelial cells (Figures 7A,B).

## Discussion

Osteoarthritis is an irreversible chronic disease that cannot be cured. With the prolongation of the disease and the worsening of symptoms, systemic pathological changes inevitably occur, which in turn affect the intestine. Functional changes in the intestine affect the pathological development of OA. In the present study, intestinal tract damage caused by OA was observed. Inflammation and oxidative stress were also present in rats. Addressing oxidative stress and inflammation is a common approach to treating OA and is currently a key research area in OA treatment (Li et al., 2022; Zhu et al., 2022). Here, we also explored the effects of probiotics on OA-induced colonic damage and found that intervention with *B. subtilis* and *E. faecium* alleviated OA-induced colonic inflammation and oxidative stress by improving fecal metabolism and enhancing the intestinal barrier.

Inflammatory factors play an important role in the pathogenesis of OA. It is a low-grade inflammatory disease, and under sustained pathological conditions, it can lead to activation of the NF-κB pathway in chondrocytes, increasing the inflammatory cytokines TNF-α, IL-1, and IL-6 (Shen et al., 2017). In OA combined with obesity, the subpatellar fat pad releases TNF-α, IL-6, IL-1, and IL-1β. TNF-α and IL-6 may indirectly contribute to OA by regulating the release of adiponectin and leptin from adipocytes (Eymard and Chevalier, 2016). This could explain the presence of excessive inflammatory factors in the serum of OA rats. Furthermore, inflammatory factors play an important role in cartilage matrix degradation and bone resorption in OA.

The mechanism underlying the effect of IL-1 on cartilage remains unclear; however, IL-1 is speculated to promote NO production by chondrocytes and induce chondrocyte apoptosis. High levels of NO can promote cartilage degradation. In contrast, IL-6 promotes the growth and differentiation of osteoclasts and increases the activity of osteoclasts, which damages the articular cartilage (Wang and He, 2018). In addition, IL-1 and IL-6 enhance the expression of MMP in chondrocytes and inhibit the synthesis of collagen and proteoglycan (Kapoor et al., 2011). The combined use of *B. subtilis* and *E. faecium* reduced cytokine production in response to inflammation (Myhill et al., 2022).

Our results also showed that *B. subtilis* and *E. faecium* can effectively reduce the pro-inflammatory factors IL-1ß and IL-6 in the serum caused by OA. However, we believe that the low level of systemic inflammation and oxidative stress present in OA leads to the disruption of intestinal function since OA development is associated with the accumulation of ROS and systemic inflammation. Reactive oxygen species regulate cytokine secretion and apoptosis of chondrocytes (Chang et al., 2020). Furthermore, elevated serum concentrations of MDA and iNOS were observed in osteoarthritic rats, with a corresponding decrease in GSH and SOD levels. The disturbance of these serum oxidative and antioxidant markers in osteoarthritic rats corresponds to intestinal dysfunction. In addition, serum concentrations of CRP, D-LAC, and DAO were significantly increased in osteoarthritic rats compared to those in normal rats. Emerging evidence suggests that gut microbes may also be considered as possible triggers of inflammation in skeletal muscle diseases such as OA (Boer et al., 2019). Therefore, manipulation of the gut microbiota may be a novel intervention to prevent OA, and the application of probiotic interventions in OA can effectively improve inflammatory factor levels (Arora et al., 2021). This result is consistent with our findings, wherein *B. subtilis* and *E. faecium* treatment significantly improved the physiological indices of the colon.

Gut microbes regulate the health of the host, while specific intestinal microbiota occurs under certain disease conditions. New evidence suggests that the intestinal microbiota plays an important role in the pathogenesis of the OA (Schott et al., 2018). This is related to systemic inflammation caused by the intestinal flora and metabolites, which affect the permeability of the intestine (Huang et al., 2020). Studies have shown that the changes in the obesity-mediated intestinal microbiome may affect low-grade systemic and local inflammation in OA (Huang and Kraus, 2016; Schott et al., 2018). Gastrointestinal microorganisms have a direct role in causing inflammation in OA. Boer et al. (2019) observed a significant correlation between the abundance of *Streptococcus* spp. and the severity of knee effusion and suggested that *Streptococcus* spp. could trigger macrophage activation through the TLR pathway, resulting in inflammation with pain. Alterations in the normal gut microbiota are associated with joint and bone diseases, such as rheumatoid arthritis and osteoporosis (Zhang et al., 2015; Li J. Y. et al., 2016).

To determine the OA-induced changes in the intestinal microbiota and the effect of probiotic treatment on flora, we assessed the status of fecal flora in all groups. Although we did not find significant differences in the α- and ß- diversity, as well as the abundance of major flora, between the three groups. However, LEfSe analysis showed the presence of *Alistipes* in osteoarthritic rats. *Alistipes* is a commensal anaerobic bacterium present in the healthy human gastrointestinal tract. As of April 2020, the NCBI classification database shows that *Alistipes* consists of 13 species. Recent studies have shown that *Alistipes* is associated with liver fibrosis, colorectal cancer, cardiovascular disease, and mood disorders (Parker et al., 2020). The discovery of this genus in OA also shows the complexity of OA pathology. *Alistipes* has a protective effect against hepatocellular carcinoma and inflammatory intestinal diseases (Li J. et al., 2016; Sung et al., 2019). However, it plays a negative role in cardiovascular disease and mood disorders. Kim et al. (2018) performed a metagenomic analysis to show that *Alistipes* may be a potential driver of intestinal barrier dysfunction and inflammation in hypertensive patients. Thus, *Alistipes* not only plays a healthy phenotypic role but also has a pathogenic role in some specific diseases and may also cause pro-inflammatory and damaging effects on the intestinal barrier (Parker et al., 2020). These studies corroborate our study results; however, different observations were also noted. We found damage to the colonic barrier and elevated inflammatory factors in the blood of osteoarthritic rats. Thus, further studies are needed to elucidate the association between *Alistipes* and OA.

Meanwhile, *Paraprevotella* was observed after *B. subtilis* and *E. faecium* treatment. *Paraprevotella* was also present in the intestinal flora of patients with depression (Jiang et al., 2015). The emergence of depressive signature bacteria indicates the severity of OA, which is a long-term irreversible injury that can cause severe mental stress to the patient or animal. Current gut interventions for OA have focused on inflammatory and pain interventions (Biver et al., 2019), with little research on psychiatric interventions for OA. This suggests the importance of psychiatric interventions for OA.

Meanwhile, analysis of fecal non-targeted metabolism showed that the fecal metabolism of the OA group was significantly different from that of the CON and TREAT groups. Histamine is a biogenic amine that acts primarily as a mediator of inflammatory and allergic reactions and is stored by mast cells and basophils. Histamine is involved in bone metabolism and can promote the differentiation of osteoblasts (Deyama et al., 2002; Yamaura et al., 2003; Biosse-Duplan et al., 2009). It is also found in the intestine, primarily in mast cells and enterochromaffin cells, and microorganisms in the intestine can produce histamines, such as *Staphylococcus* and some bacteria of the *Enterobacteriaceae* (Sanchez-Perez et al., 2022). In the gastrointestinal tract, histamine levels can be influenced by inflammatory responses, degradative enzymes, and gut microbes. Histamine acts as a pro- and anti-inflammatory agent in the gut, depending on which class of histamine receptors it activates (Smolinska et al., 2014). Our study showed a decrease in histamine levels in the OA gut, which may be related to elevated serum levels of DAO and damage to the intestinal barrier. The activity of DAO is negatively correlated with histamine (Pinzer et al., 2018). Elevated intestinal permeability contributes to the penetration of histamine produced by gut microbes (Sanchez-Perez et al., 2022).

Osteoarthritis development is accompanied by systemic low-grade inflammation. Under inflammatory conditions, mitochondrial dysfunction and ROS production are inevitable. The OA and CON groups had different expression levels of pantothenate and CoA biosynthesis, and ß-alanine metabolism pathways. Pantothenate is a precursor of CoA, which is involved in the TCA cycle. In contrast, differences in the ß-alanine metabolism pathway indicate differences in protein mobilization by the organism. ß-Alanine is involved in the metabolism of carnosine (Derave et al., 2010). These results indicate a significant difference in energy metabolism between the OA and CON groups. Under inflammatory conditions, chondrocytes undergo pathological changes in metabolic homeostasis and cartilage remodeling, characterized by enhanced glycolytic pathways and mitochondrial dysfunction (Mobasheri et al., 2017). This is consistent with our findings wherein increased levels of serum pro-inflammatory factors in osteoarthritic rats lead to increased levels of MDA and iNOS. This suggests that the local pathological changes in OA have affected the whole body.

Enhanced anaerobic enzymolysis in OA leads to energy deficiency in the organism, as has been shown by the differential metabolic pathways annotated to intestinal metabolites. Although treatment with *B. subtilis* and *E. faecium* did not improve histamine levels in the intestine of osteoarthritic rats, linoleic acid metabolism, fatty acid biosynthesis, and primary bile acid biosynthesis were observed compared to that in the CON group. These pathways are associated with energy metabolism, anti-inflammatory properties, and antioxidant properties. Therefore, *B. subtilis* and *E. faecium* can interfere with the inflammation and oxidative stress status of osteoarthritic rats. Between the TREAT and OA groups, all differential metabolites were found to be downregulated except for taurocholate, palmitic acid, and cyclohexylamine metabolites, which were upregulated. L-glutamate, which is derived from the glutamine-catalyzed conversion of glutamine by glutaminase (Altman et al., 2016), was downregulated. Glutamate is essential for intracellular GHS synthesis and maintains the cellular redox homeostasis (Xiang et al., 2013). Sayin et al. (2017) found that tumor cells increase their antioxidant activity by increasing glutamate uptake to synthesize GSH through excessive activation of the Nrf2 pathway. This is our observation that the expression of Nrf2 protein increased in colonic tissues after *B. subtilis* and *E. faecium* treatment. Meanwhile, non-targeted metabolism indicated a decrease in the amount of L-glutamate in the colon, and the level of GSH in the serum was increased in the TREAT group, suggesting that *B. subtilis* and *E. faecium* acted as antioxidants in the intestine by promoting the consumption of L-glutamate.

The metabolic pathway central carbon metabolism in cancer differed from OA when treated with *B. subtilis* and *E. faecium*, demonstrating that energy metabolism was different from that of the OA group. Other differential metabolic pathways annotated were FoxO signaling pathway, Huntington's disease, spinocerebellar ataxia, long-term potentiation, and taurine and hypotaurine metabolism. These results indicate the differences in skeletal muscle performance, spatial cognition, and oxidative stress in the TREAT group compared to those in the OA group (Akasaki et al., 2014; Berggren et al., 2015; Thirupathi et al., 2018; Agarwal et al., 2021). In summary, we found that *B. subtilis* and *E. faecium* can affect oxidative stress in the colon of OA rats.

We then further analyzed the differences in protein expression. Previously, we found differences between serum pro-inflammatory, oxidative, and antioxidant factors in osteoarthritic rats. We further examined the protein expression on the oxidative stress-related pathway Keap1/Nrf2 in colonic tissue. Nrf2 is the main transcription factor, and Keap1 is the molecular sensor for reactive species. At normal conditions, Keap1 fixes Nrf2, leading to Nrf2 ubiquitination and proteasomal degradation; therefore, Nrf2 abundance is low. Under oxidative stress conditions, Keap1 is modified on some specific cysteine moieties, which disables the activity of the E3 ligase adaptor. As a result, the Nrf2 level is increased. When Nrf2 exceeds the levels of Keap1, Nrf2 will escape the fixation by Keap1 and translocate into the nucleus, wherein it combines with the small musculoaponeurotic fibrosarcoma (sMaf) proteins to form Nrf2-sMaf heterodimer. The heterodimer recognizes the antioxidant response elements in the promoters of target genes to induce transcription, then performs the antioxidant functions (Liu et al., 2022). We found that the Keap1/Nrf2 pathway was inhibited in the colon of osteoarthritic rats, while *B. subtilis* and *E. faecium* treatment activated the pathway and increased the entry of Nrf2 into the nucleus. Activation of the Keap1/Nrf2 pathway was accompanied by a corresponding increase in the relative expression of the antioxidant-protective proteins HO-1 and Gpx4. Gpx4 is associated with lipid peroxidation, the presence of which has been observed in the OA (Miao et al., 2022). We hypothesized that the antioxidant protection in the colonic tissue of osteoarthritic rats by *B. subtilis* and *E. faecium* is related to the integrity of the colonic barrier. The histological, immunohistochemical, and mRNA results corroborated our hypothesis. *B. subtilis* and *E. faecium* treatment significantly ameliorated the damage to the colon tissue and reduced the relative expression of the apoptosis-associated protein Bax, which effectively prevented LPS from entering the bloodstream, thereby reducing the inflammatory and oxidative stress state of the organism (Zhang et al., 2021).

Many studies demonstrate the link between osteoarthritis and the gut, which is a target osteoarthritis treatment (Schott et al., 2018; Lian et al., 2022). The relationship between intestinal injury caused by OA and intestinal injury inversely affecting the pathological process of OA is extremely complex and needs further research. Furthermore, our present study has limitations. First, the number of samples in our study was relatively small. Second, tissue metabolism, joint fluid, and blood of osteoarthritic rats were unknown. If the metabolic spectrum is elucidated, evidence of the promoting effect of *B. subtilis* and *E. faecium* treatment will be more robust. Third, the duration of this experiment was relatively short, which could only reflect the early onset stage of OA. For this long-term chronic disease, how the colon environment changes over time and the intervention effects of functional probiotics need to be further explored.

## Conclusion

Oral administration of combined live *B. subtilis* and *E. faecium* effectively alleviated inflammation in the colon of OA rats by improving fecal metabolism, enhancing the colonic barrier, activating the Keap1/Nrf2 pathway, promoting Nrf2 entry into the nucleus, and upregulating the antioxidant proteins HO-1 and Gpx4 to resist OA-induced colonic oxidative stress.

## Data availability statement

The 16S rRNA datasets prented in this study can be found in online repositories. The link is https://www.ncbi.nlm.nih.gov/, and the accession number is SRP332342:PRJNA754153.

## Ethics statement

The Laboratory Animal Welfare and Ethics Committee of Northeast Agricultural University approved the animal experiment and experiment design in this study (#NEAU-2021-05-0254-7).

## Author contributions

JT, XS, MZ, and HC performed the study. JT, YW, BZ, SY, and TM performed the statistical analysis and drafted the manuscript. LG reviewed the manuscript. All authors contributed to the manuscript and approved the submitted version.

## Funding

This work was supported by the National Key R&D Program of China (2017YFD0502200), the Applied Technology Research and Development Plan of Heilongjiang (GX18B023), the Inner Mongolia Autonomous Region Science and Technology Plan Project (2022YFSH0052), and Xingjiang Uygur Autonomous Region Science and Technology Support Project Plan (2020E0236).

## Conflict of interest

The authors declare that the research was conducted in the absence of any commercial or financial relationships that could be construed as a potential conflict of interest.

## Publisher's note

All claims expressed in this article are solely those of the authors and do not necessarily represent those of their affiliated organizations, or those of the publisher, the editors and the reviewers. Any product that may be evaluated in this article, or claim that may be made by its manufacturer, is not guaranteed or endorsed by the publisher.

## Footnotes

^1^ https://docs.qiime2.org/2019.4/tutorials/
