# Supplementary figures and images for: Oral administration of live combined Bacillus subtilis and Enterococcus faecium alleviates colonic oxidative stress and inflammation in osteoarthritic rats by improving fecal microbiome metabolism and enhancing the colonic barrier
Source: Front Microbiol. 2022 Nov 10;13:1005842. doi: 10.3389/fmicb.2022.1005842 (PMC9686382; doi:10.3389/fmicb.2022.1005842)

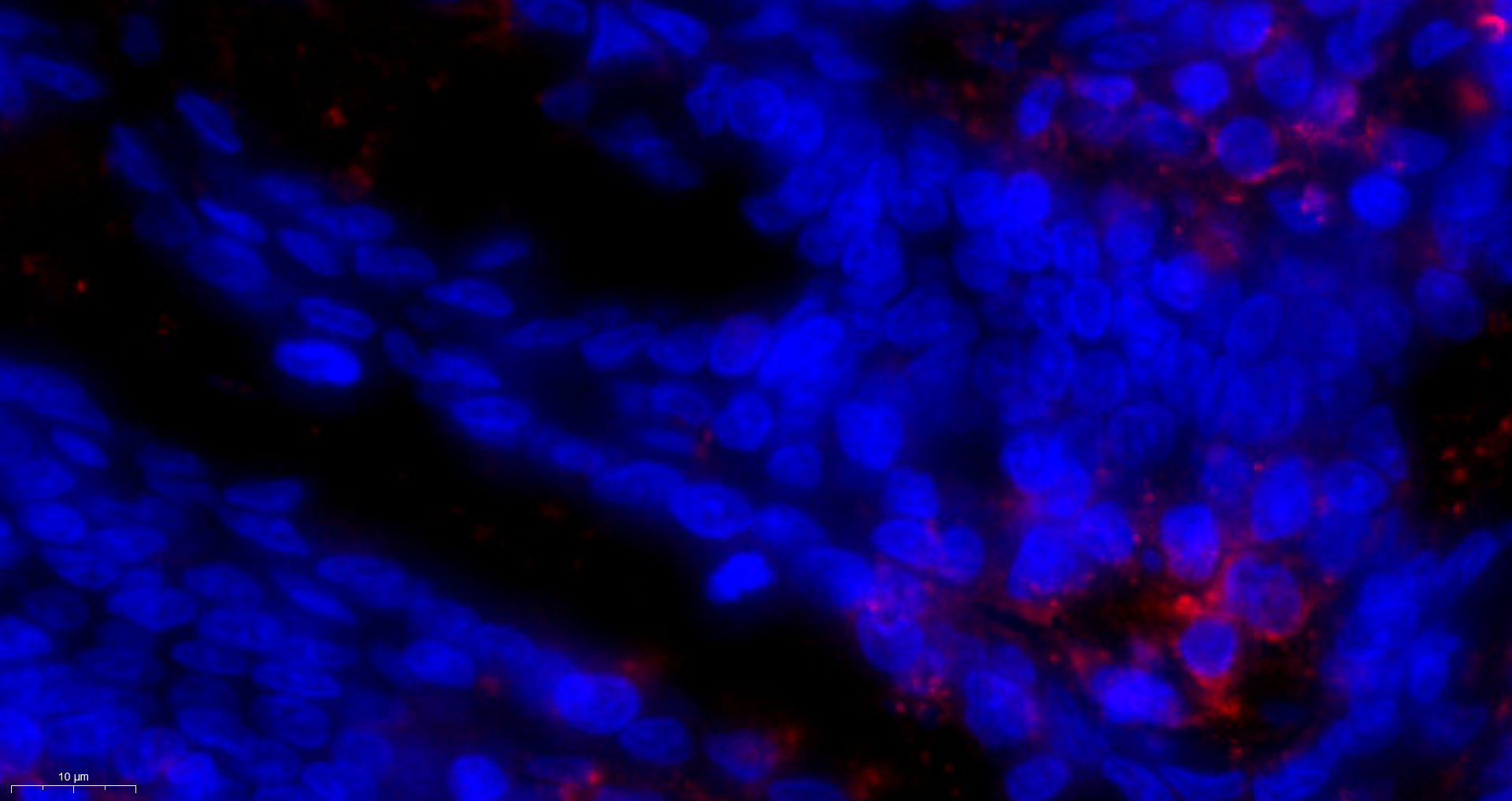

Supplement: Supplementary file 1 [file Data_Sheet_1.ZIP › raw data/Immunofluorescence/OA NRF2 red_157.8xNrf2 entering the nucleus.tif]

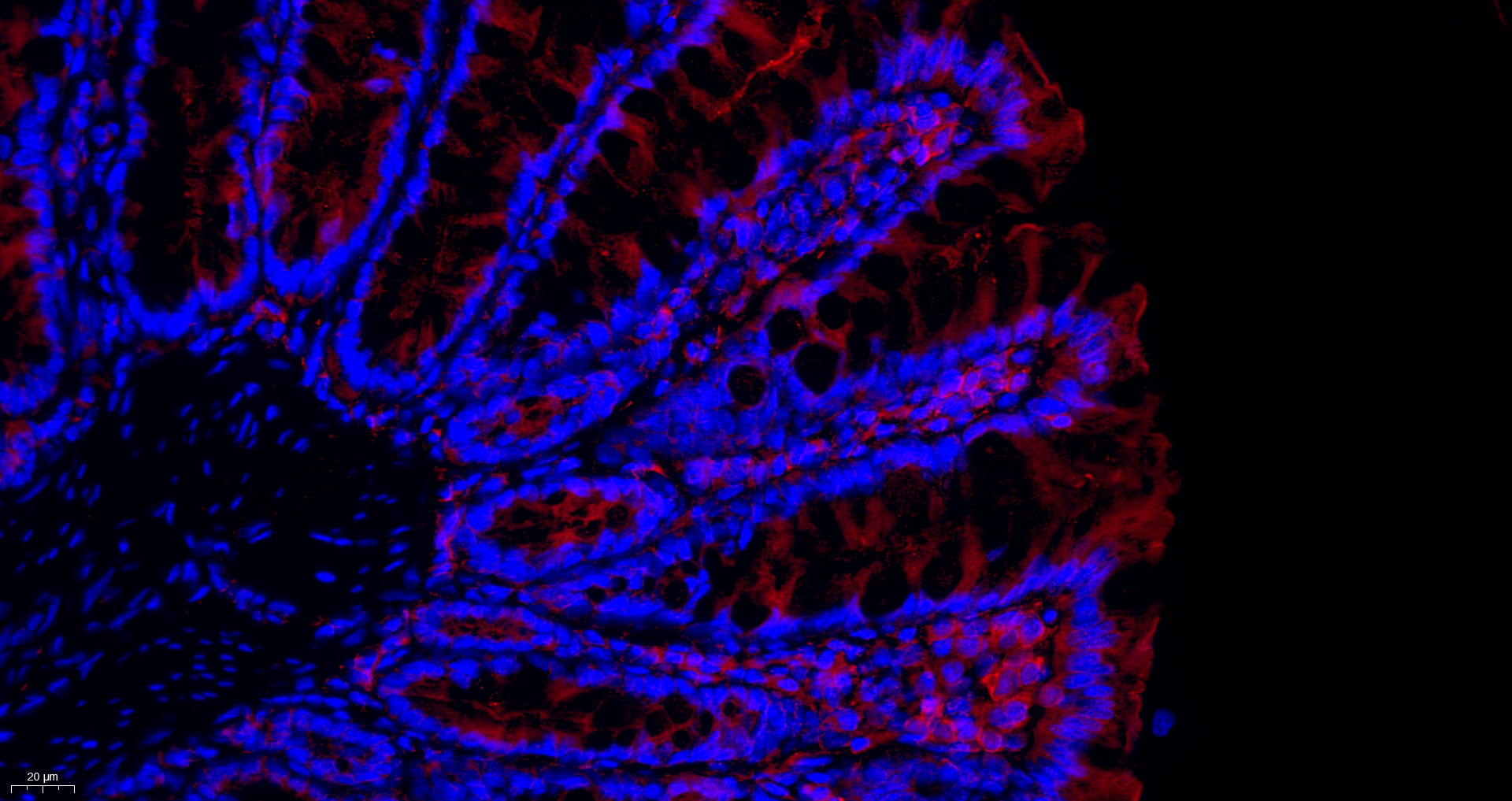

Supplement: Supplementary file 1 [file Data_Sheet_1.ZIP › raw data/Immunofluorescence/CON NRF2 red_40.0x.tif]

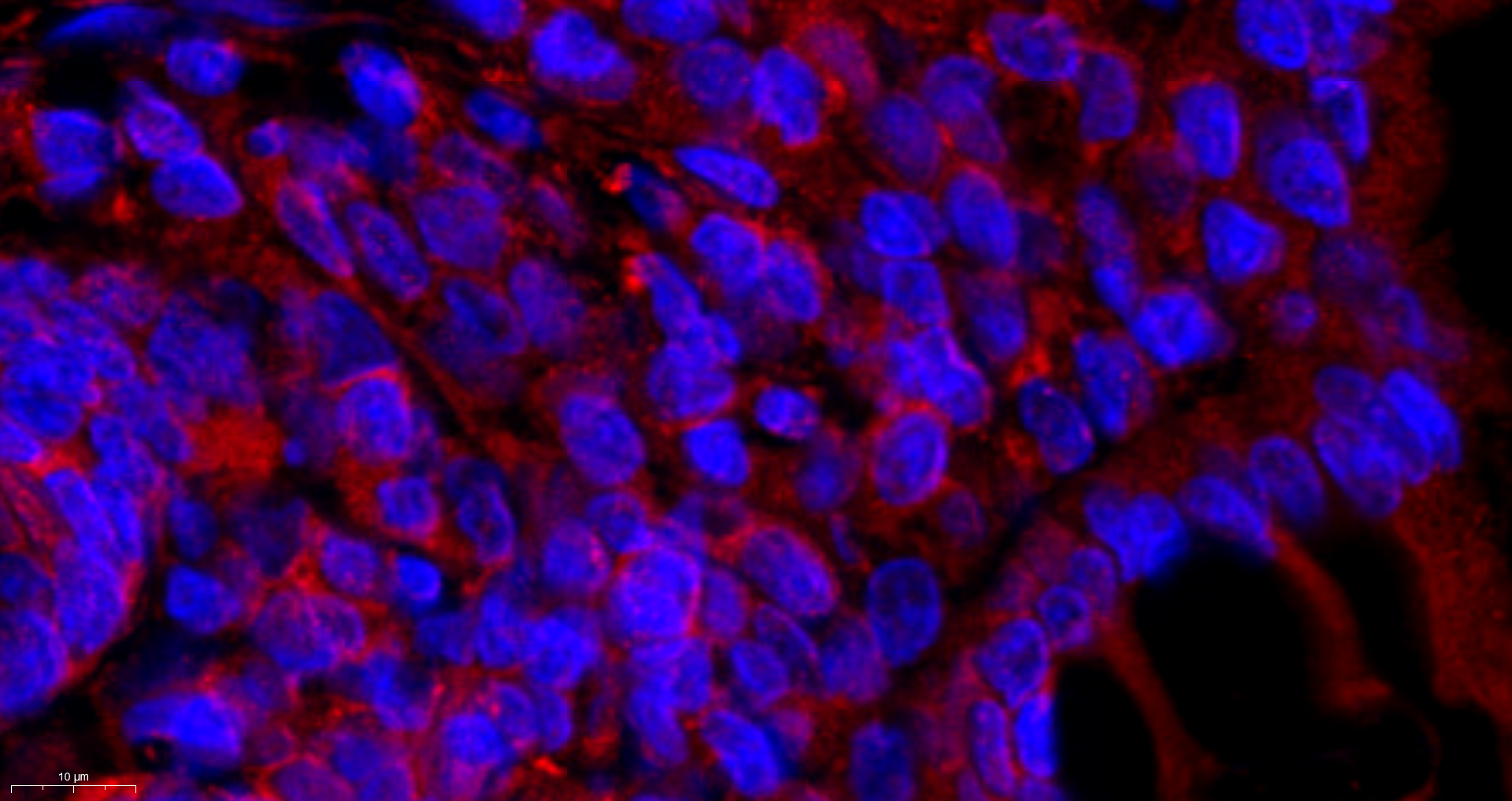

Supplement: Supplementary file 1 [file Data_Sheet_1.ZIP › raw data/Immunofluorescence/TREAT NRF2 red_157.8xNrf2 entering the nucleus.tif]

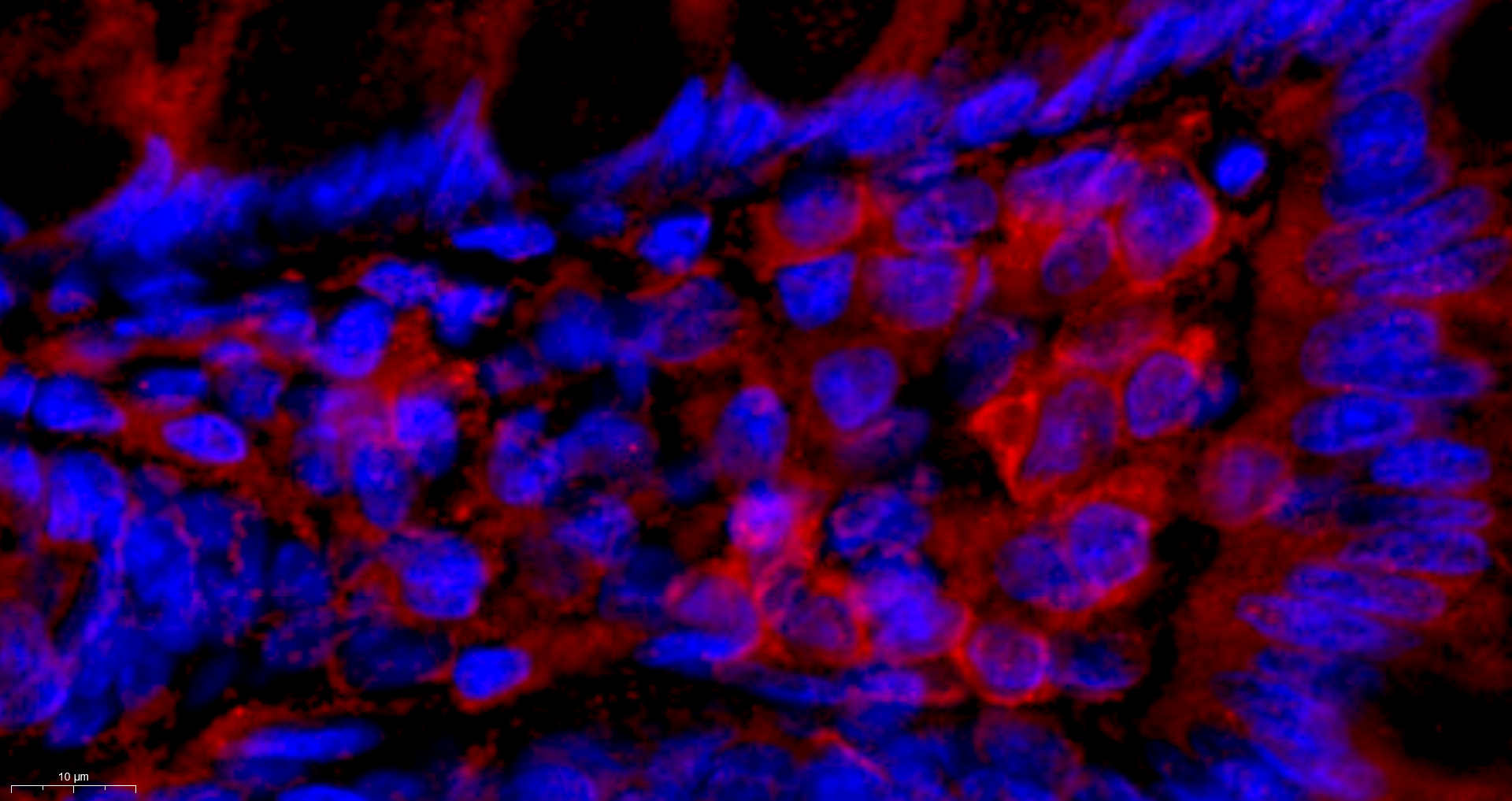

Supplement: Supplementary file 1 [file Data_Sheet_1.ZIP › raw data/Immunofluorescence/CON NRF2 red_157.8xNrf2 entering the nucleus.tif]

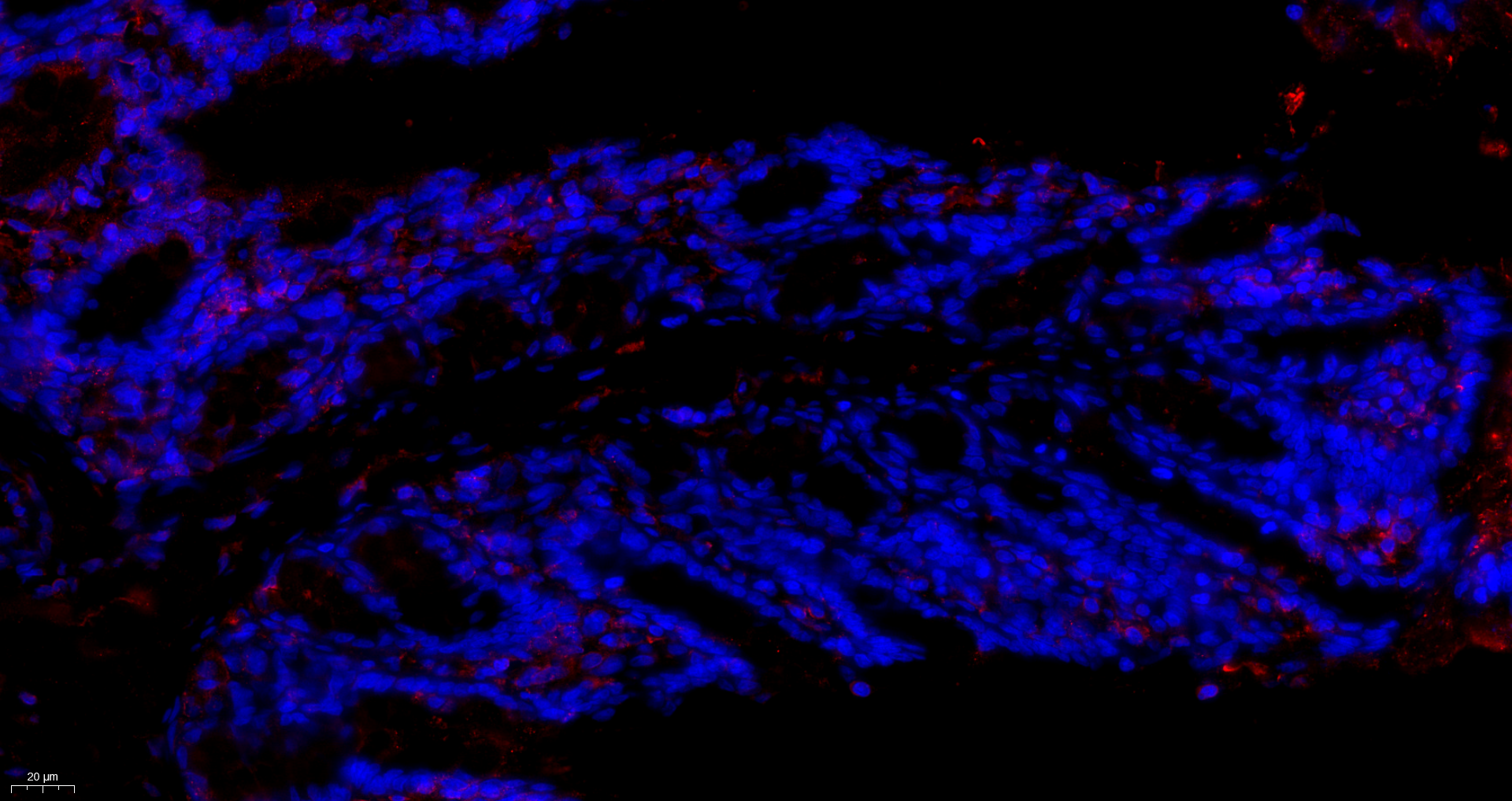

Supplement: Supplementary file 1 [file Data_Sheet_1.ZIP › raw data/Immunofluorescence/OA NRF2 red_40.0x.tif]

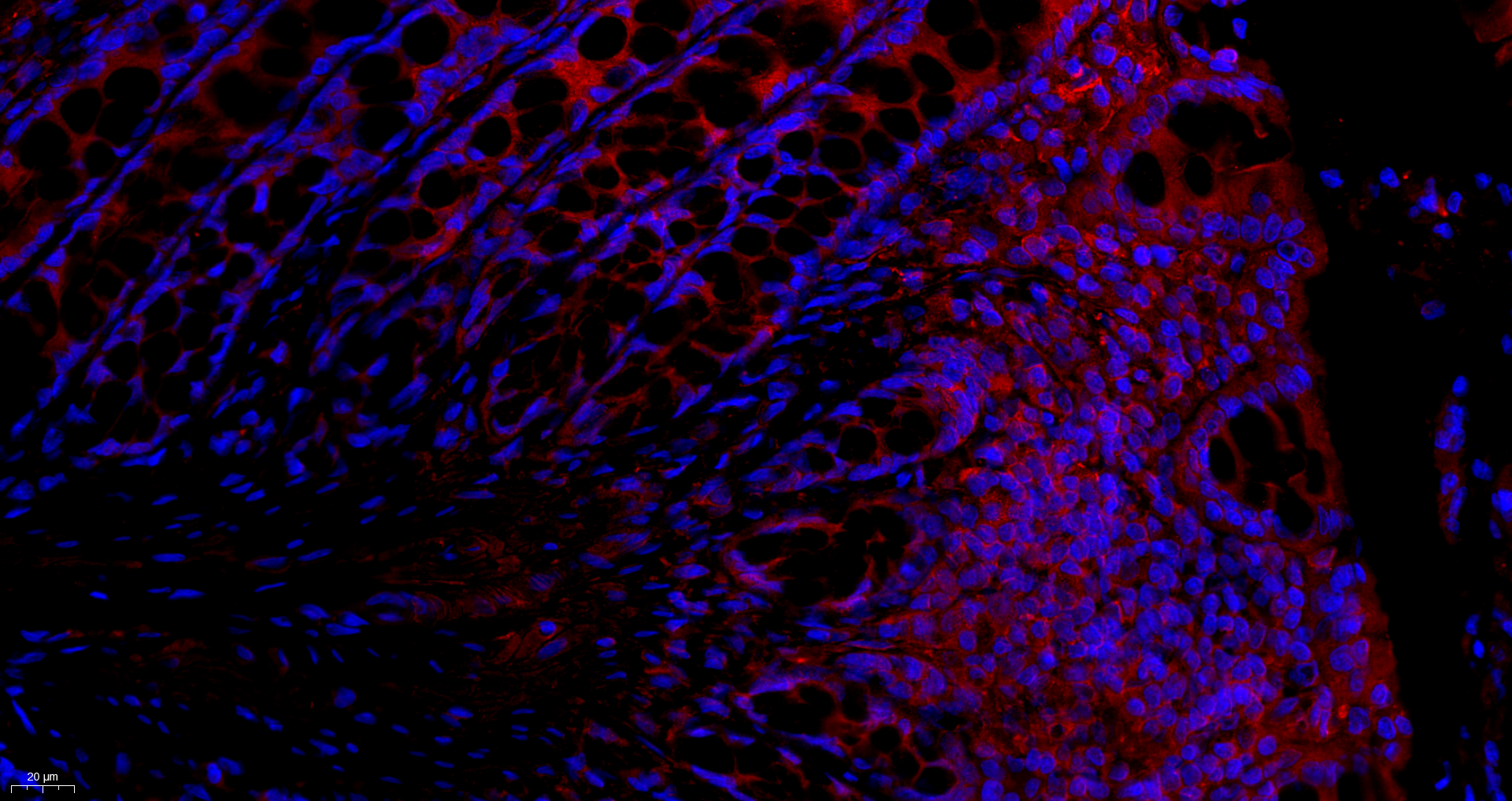

Supplement: Supplementary file 1 [file Data_Sheet_1.ZIP › raw data/Immunofluorescence/TREAT NRF2 red_40.0x.tif]

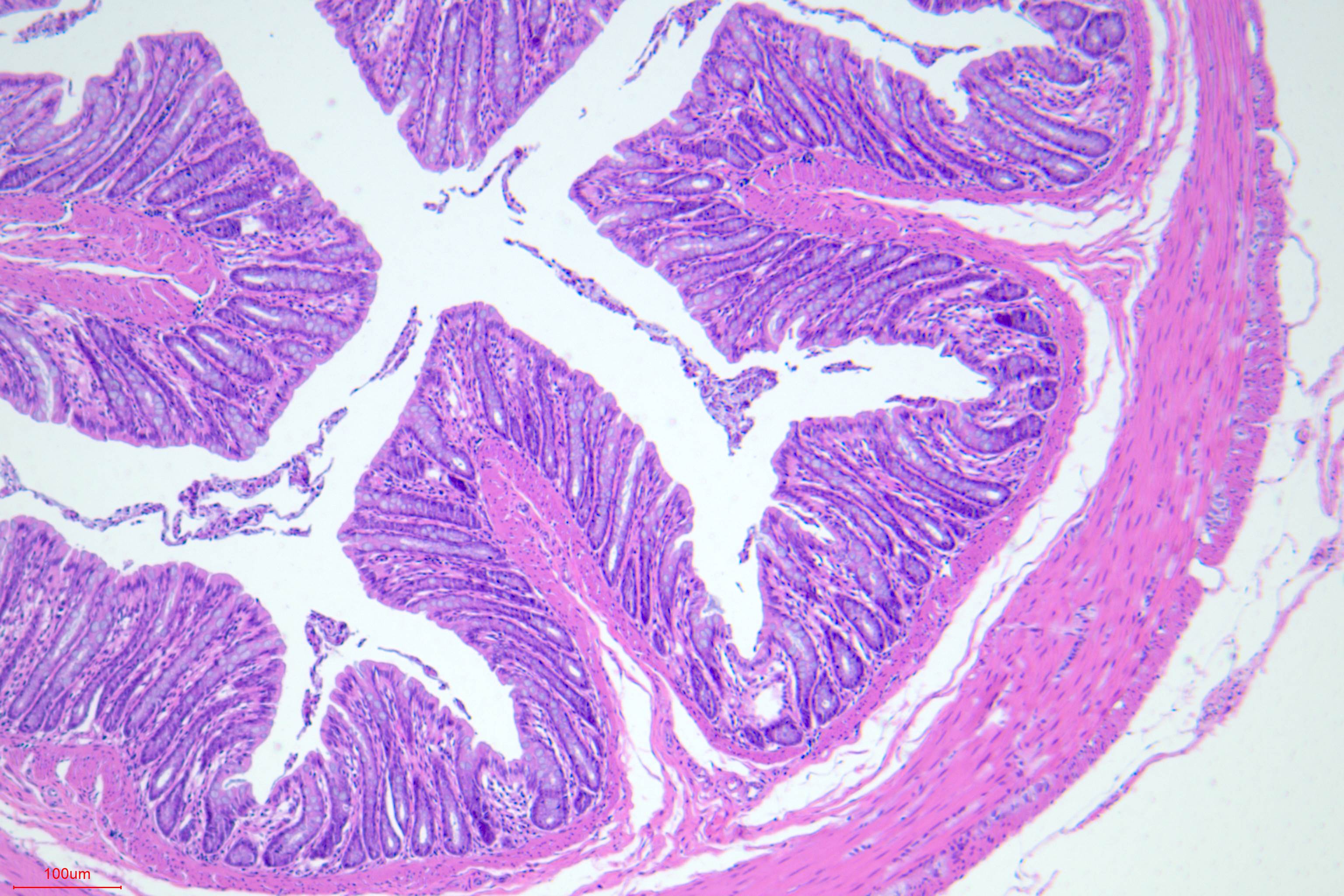

Supplement: Supplementary file 1 [file Data_Sheet_1.ZIP › raw data/H.E staning/TREAT.jpg]

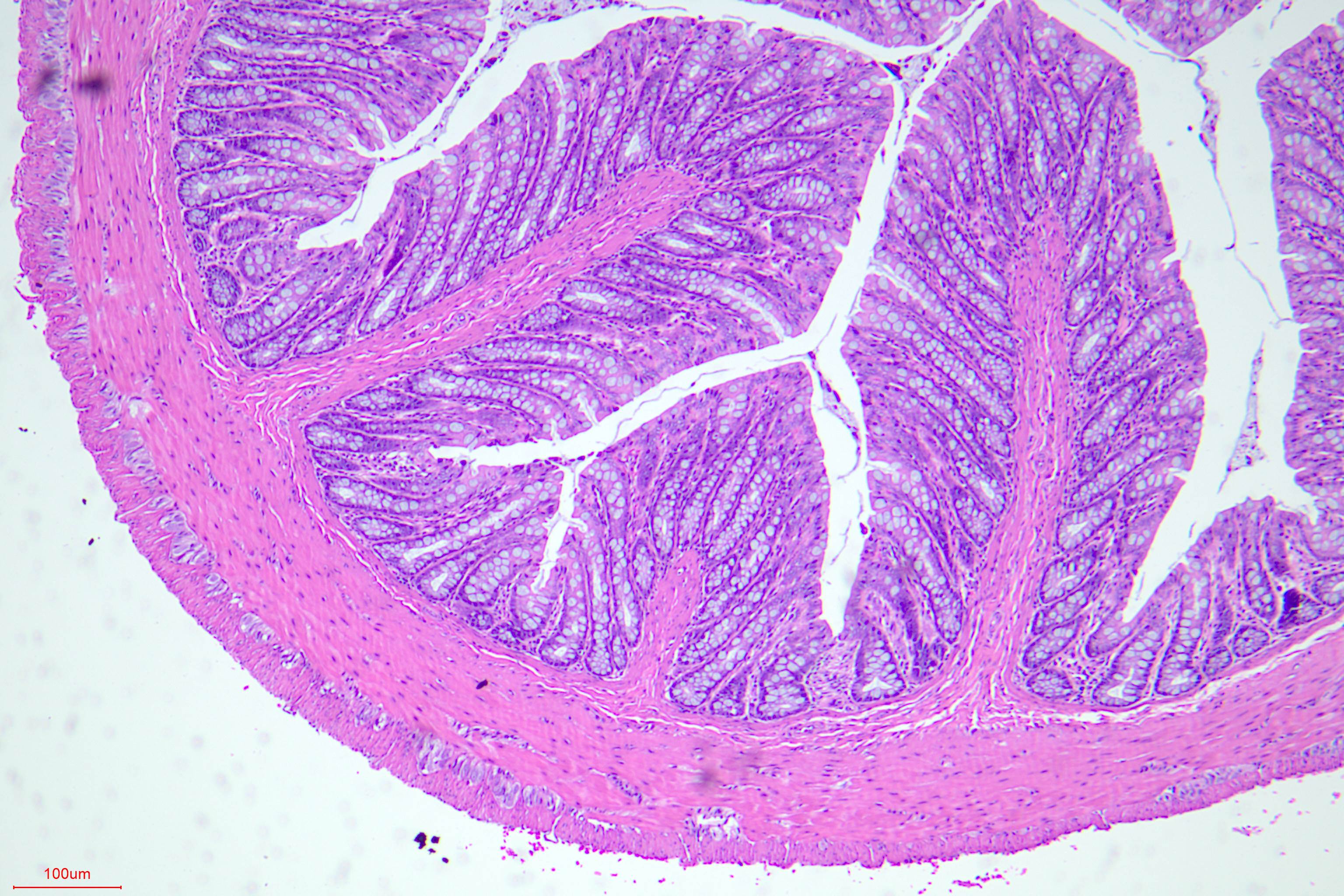

Supplement: Supplementary file 1 [file Data_Sheet_1.ZIP › raw data/H.E staning/CON.jpg]

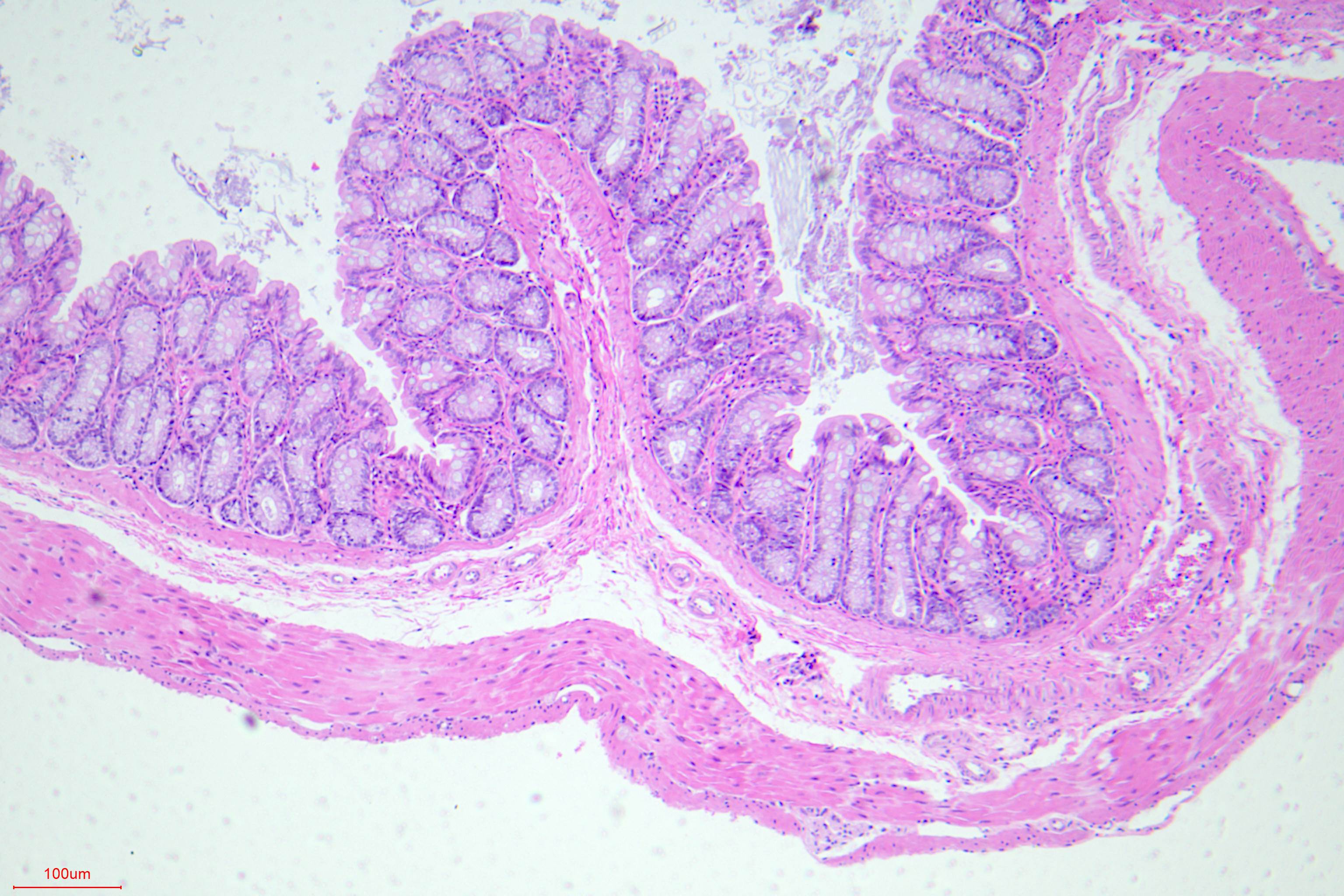

Supplement: Supplementary file 1 [file Data_Sheet_1.ZIP › raw data/H.E staning/OA.jpg]

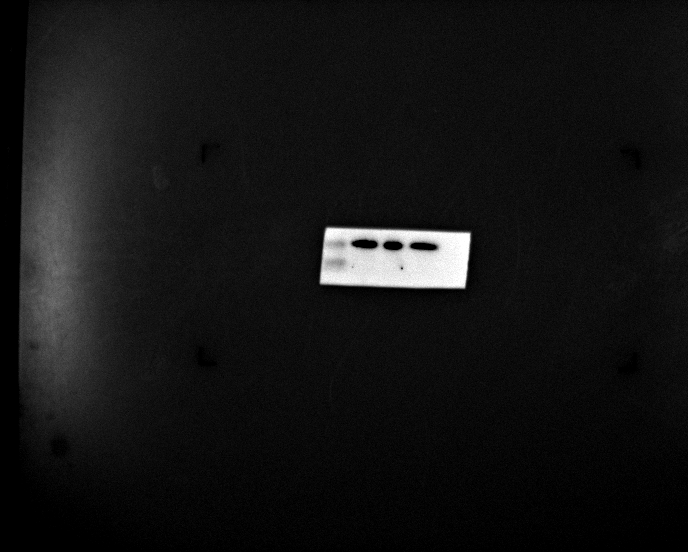

Supplement: Supplementary file 1 [file Data_Sheet_1.ZIP › raw data/western blot/Gpx4/Gpx4,1.tif]

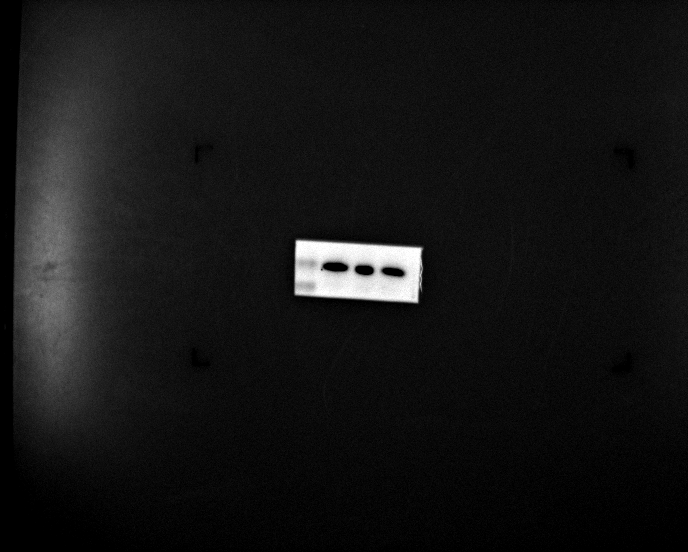

Supplement: Supplementary file 1 [file Data_Sheet_1.ZIP › raw data/western blot/Gpx4/Gpx4,2.tif]

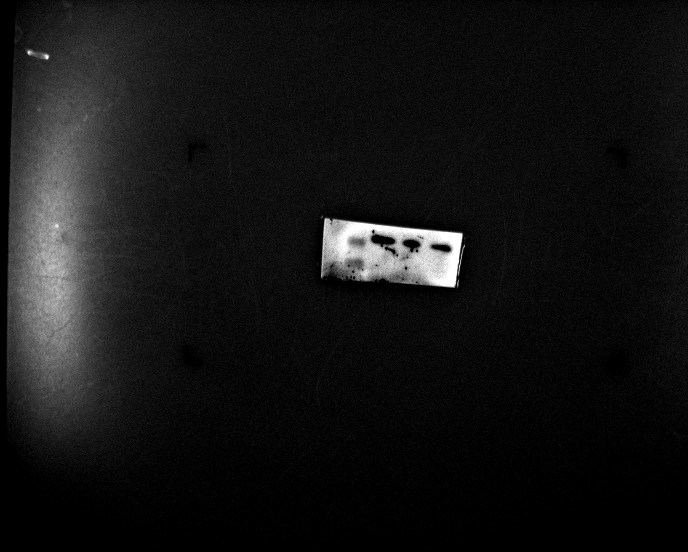

Supplement: Supplementary file 1 [file Data_Sheet_1.ZIP › raw data/western blot/Gpx4/Gpx4,3.tif]

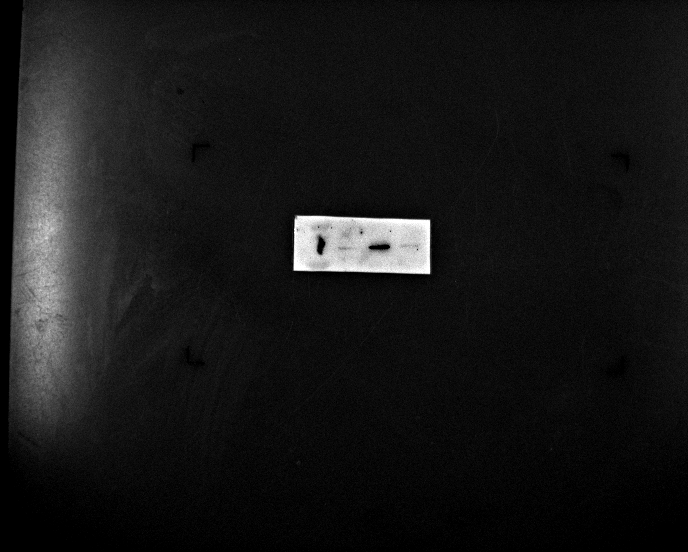

Supplement: Supplementary file 1 [file Data_Sheet_1.ZIP › raw data/western blot/Bax/Bax,1.tif]

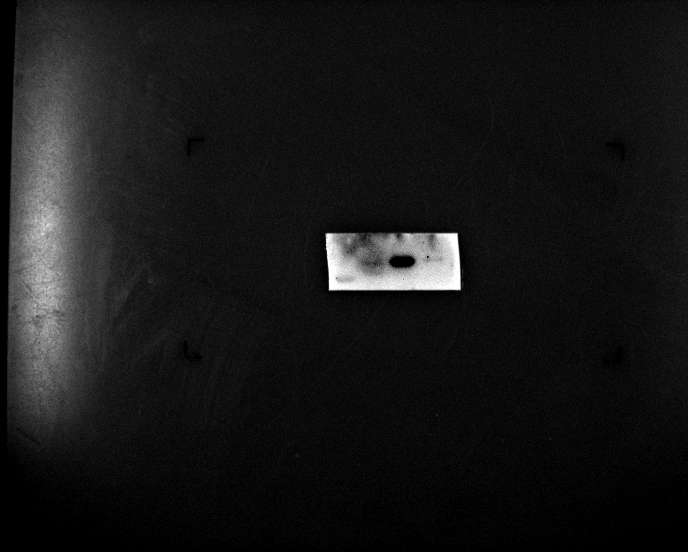

Supplement: Supplementary file 1 [file Data_Sheet_1.ZIP › raw data/western blot/Bax/Bax,2.tif]

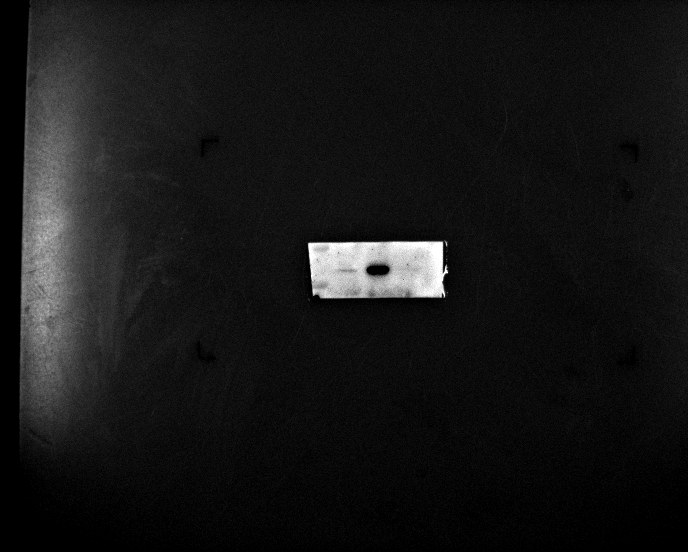

Supplement: Supplementary file 1 [file Data_Sheet_1.ZIP › raw data/western blot/Bax/Bax,3.tif]

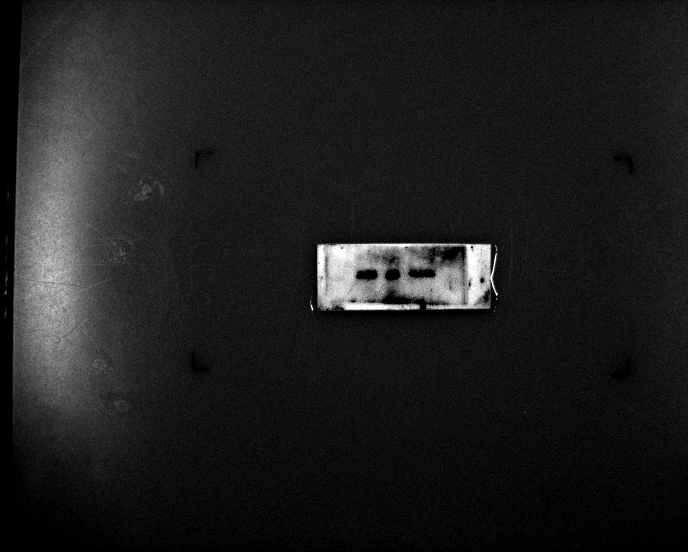

Supplement: Supplementary file 1 [file Data_Sheet_1.ZIP › raw data/western blot/HO-1/HO-1,2.tif]

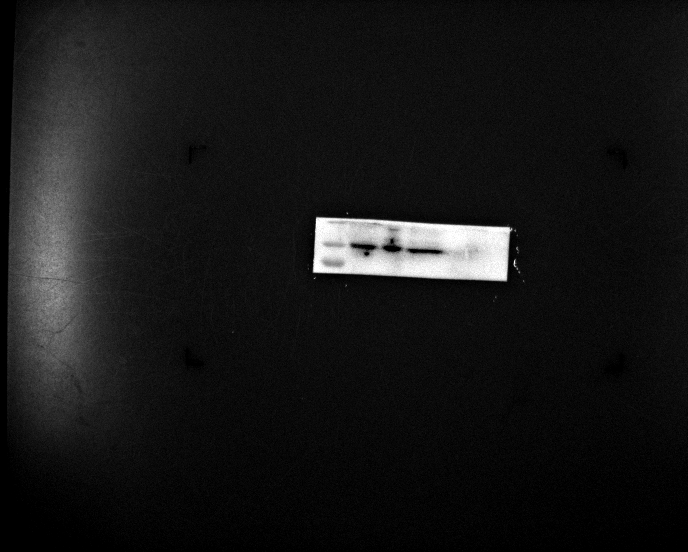

Supplement: Supplementary file 1 [file Data_Sheet_1.ZIP › raw data/western blot/HO-1/HO-1,3.tif]

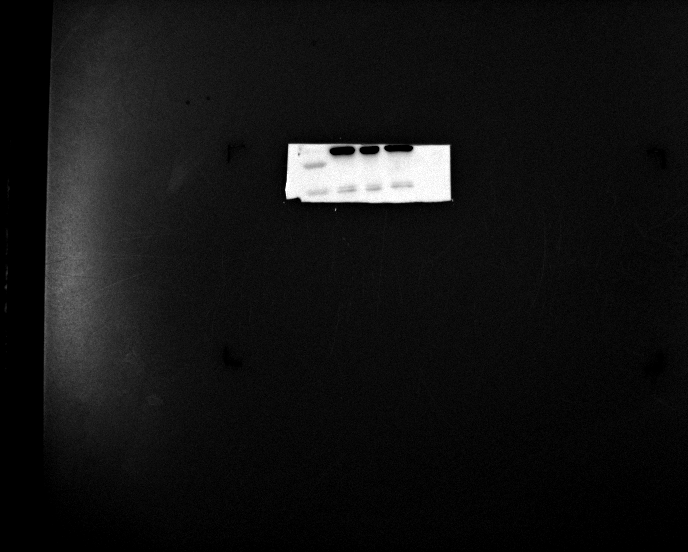

Supplement: Supplementary file 1 [file Data_Sheet_1.ZIP › raw data/western blot/HO-1/HO-1,1.tif]

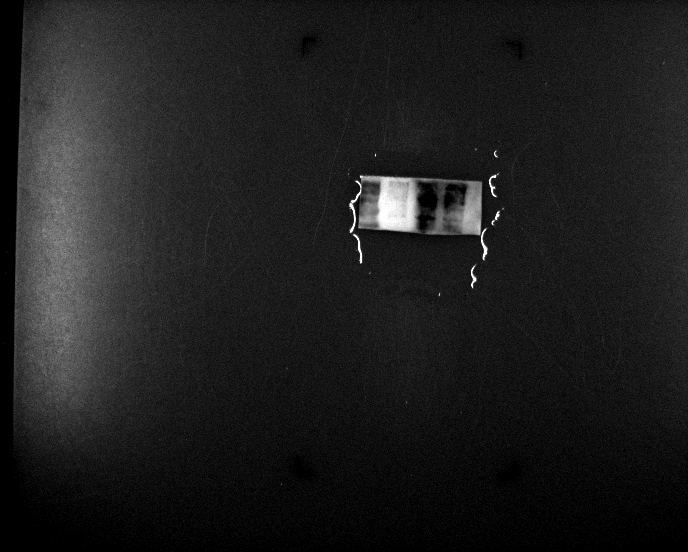

Supplement: Supplementary file 1 [file Data_Sheet_1.ZIP › raw data/western blot/Keap1/Keap1,3.tif]

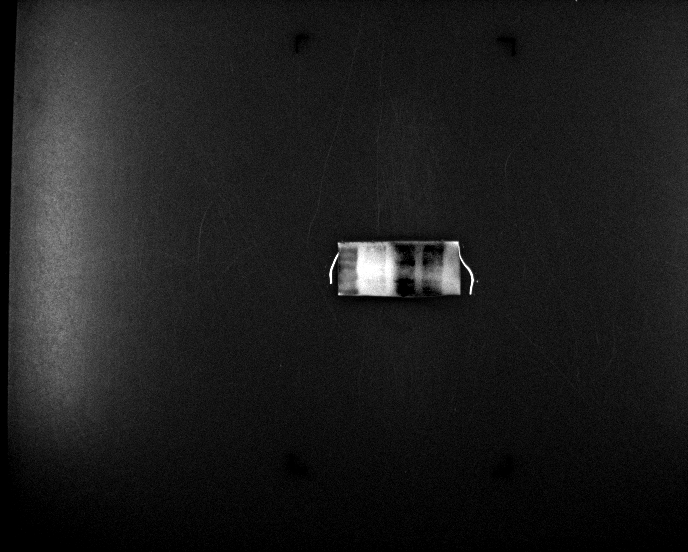

Supplement: Supplementary file 1 [file Data_Sheet_1.ZIP › raw data/western blot/Keap1/Keap1,2.tif]

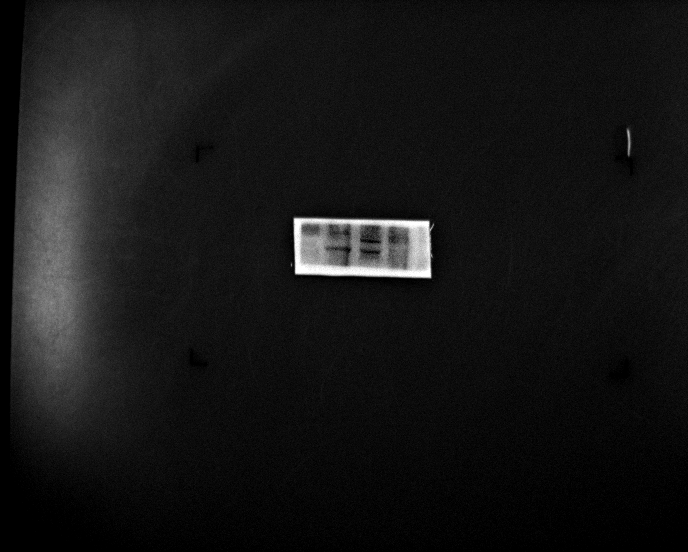

Supplement: Supplementary file 1 [file Data_Sheet_1.ZIP › raw data/western blot/Keap1/Keap1,1.tif]

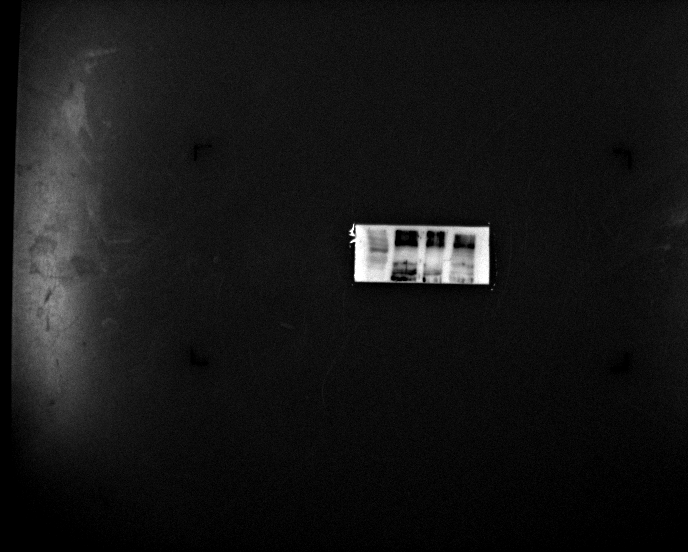

Supplement: Supplementary file 1 [file Data_Sheet_1.ZIP › raw data/western blot/Nrf2/Nrf2,2.tif]

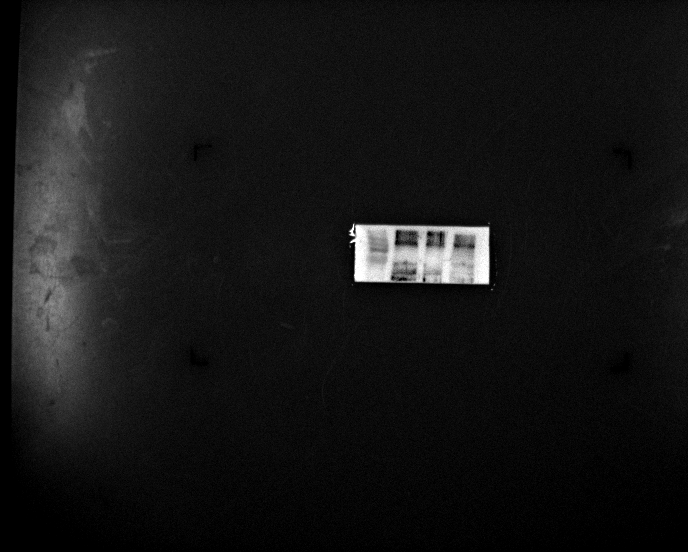

Supplement: Supplementary file 1 [file Data_Sheet_1.ZIP › raw data/western blot/Nrf2/Nrf2,3.tif]

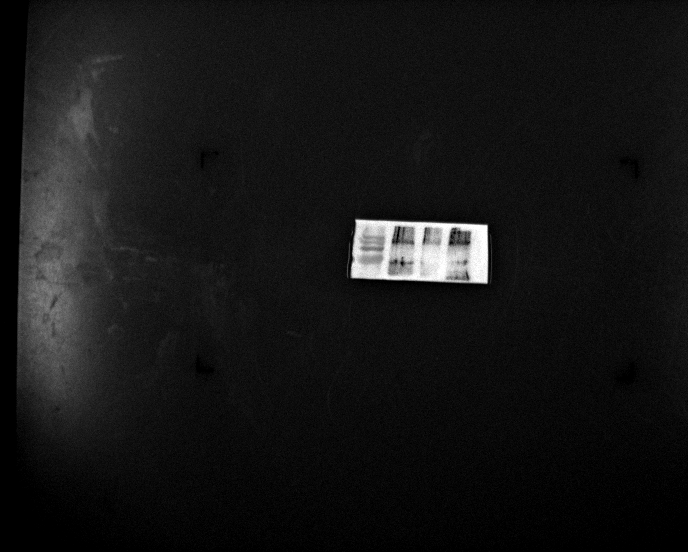

Supplement: Supplementary file 1 [file Data_Sheet_1.ZIP › raw data/western blot/Nrf2/Nrf2,1.tif]

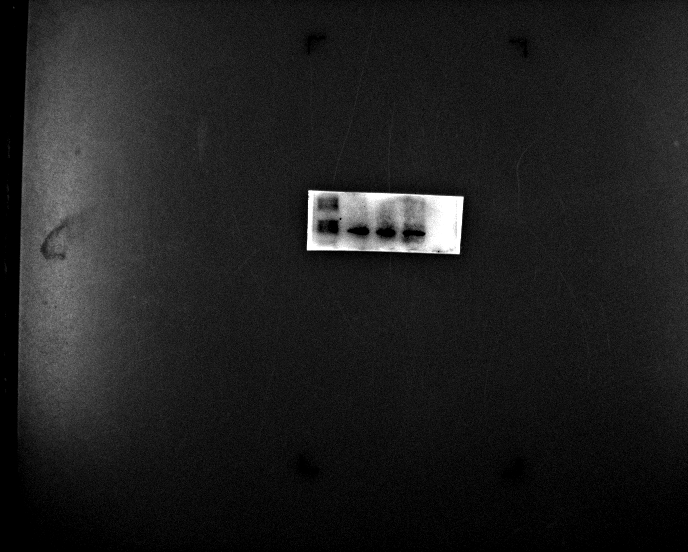

Supplement: Supplementary file 1 [file Data_Sheet_1.ZIP › raw data/western blot/├ƒ-actin/├ƒ-actin2.tif]

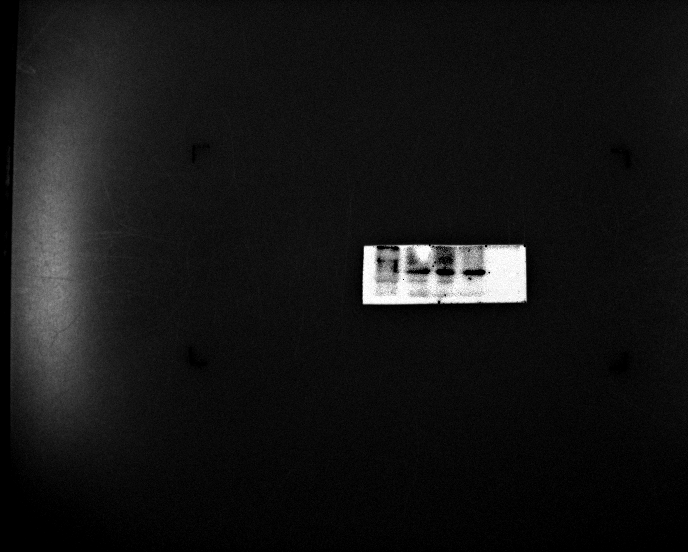

Supplement: Supplementary file 1 [file Data_Sheet_1.ZIP › raw data/western blot/├ƒ-actin/├ƒ-actin3.tif]

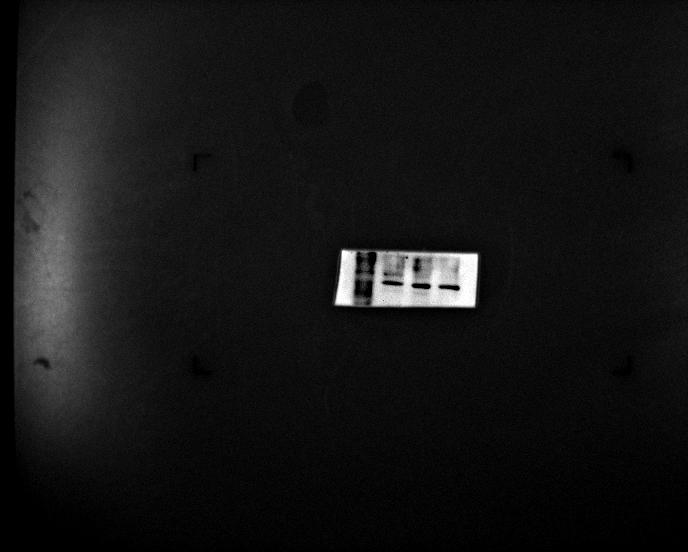

Supplement: Supplementary file 1 [file Data_Sheet_1.ZIP › raw data/western blot/├ƒ-actin/├ƒ-actin1.tif]

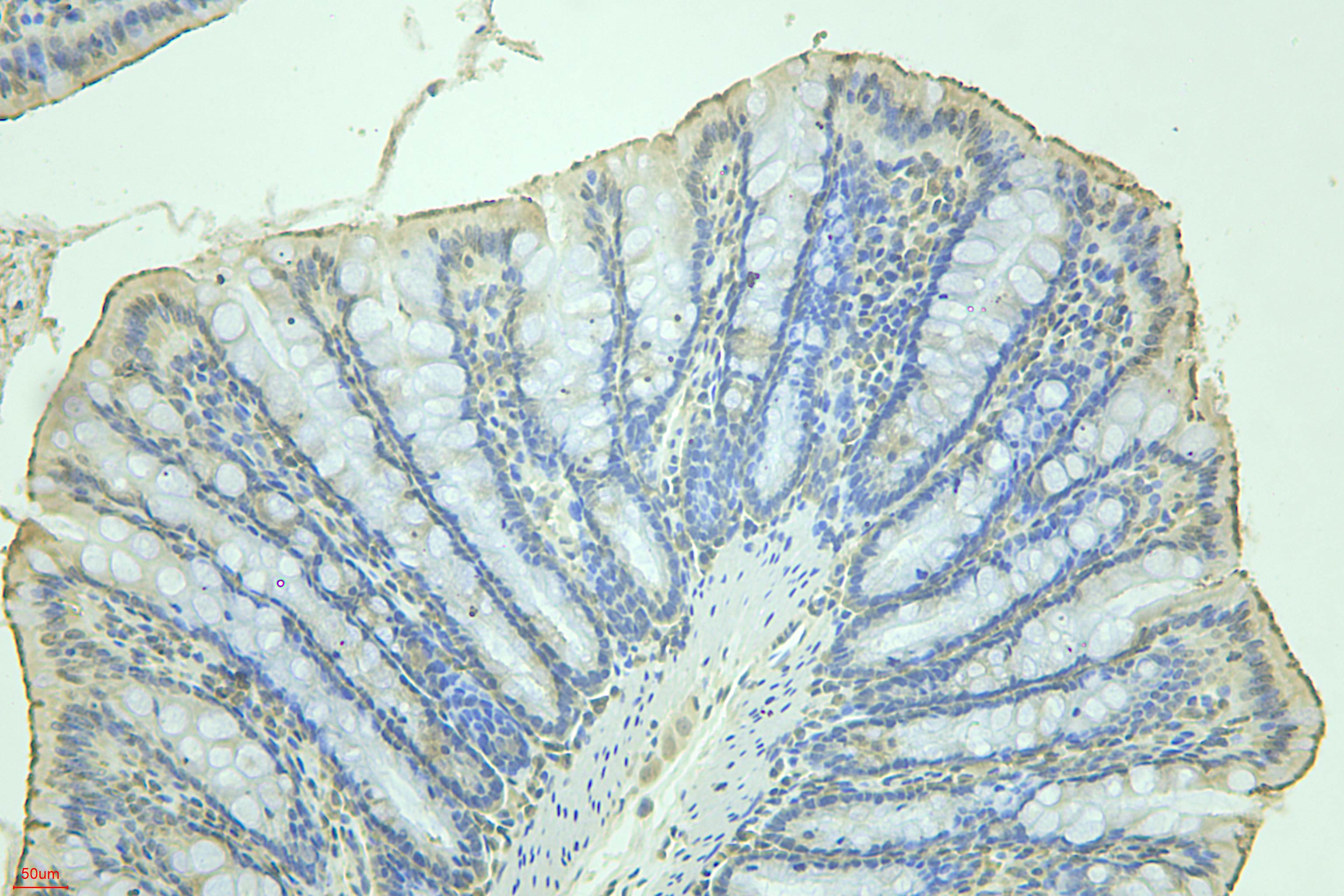

Supplement: Supplementary file 1 [file Data_Sheet_1.ZIP › raw data/Immunohistochemistry/Occludin/CON Occludin 100.jpg]

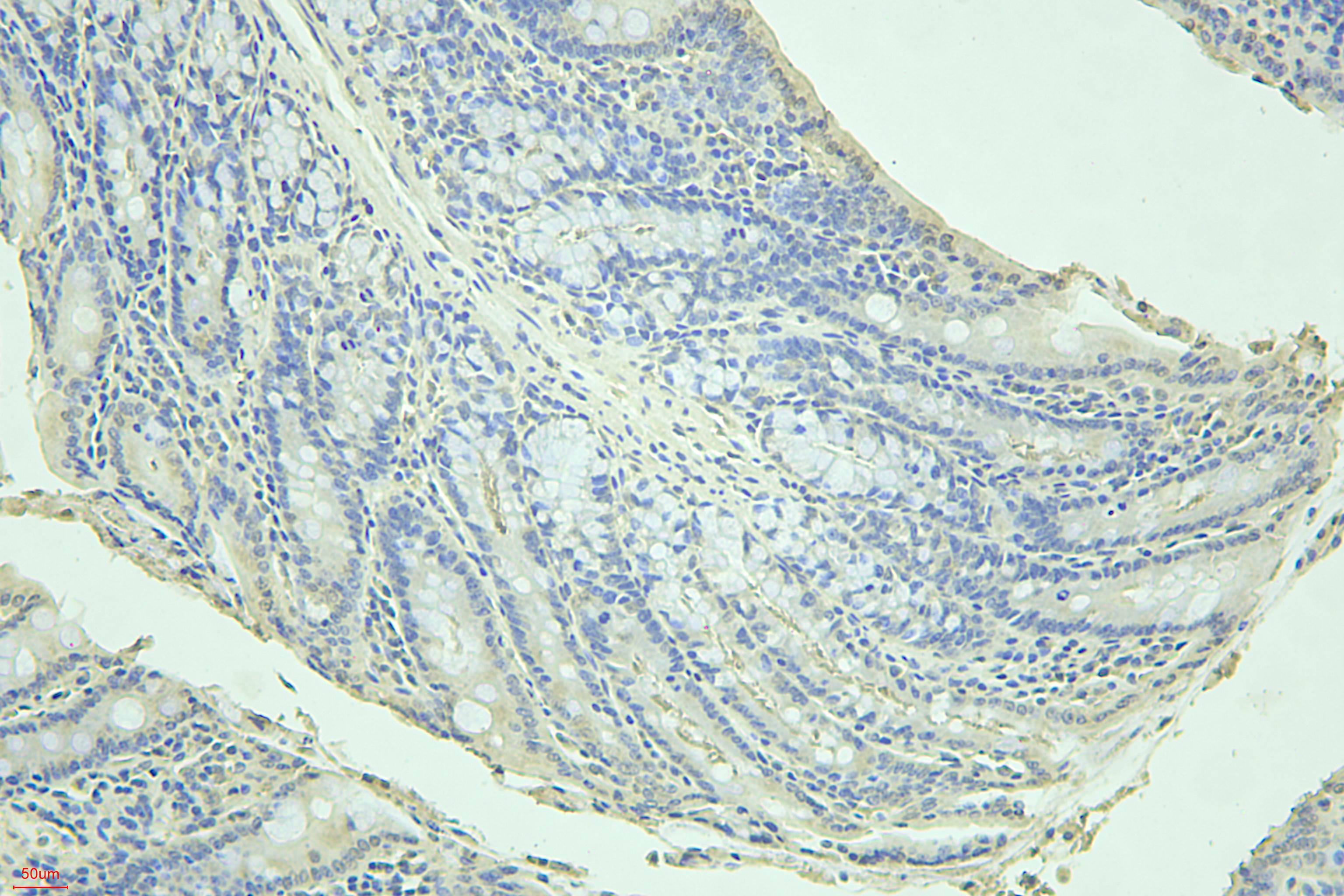

Supplement: Supplementary file 1 [file Data_Sheet_1.ZIP › raw data/Immunohistochemistry/Occludin/TREAT Occludin 100.jpg]

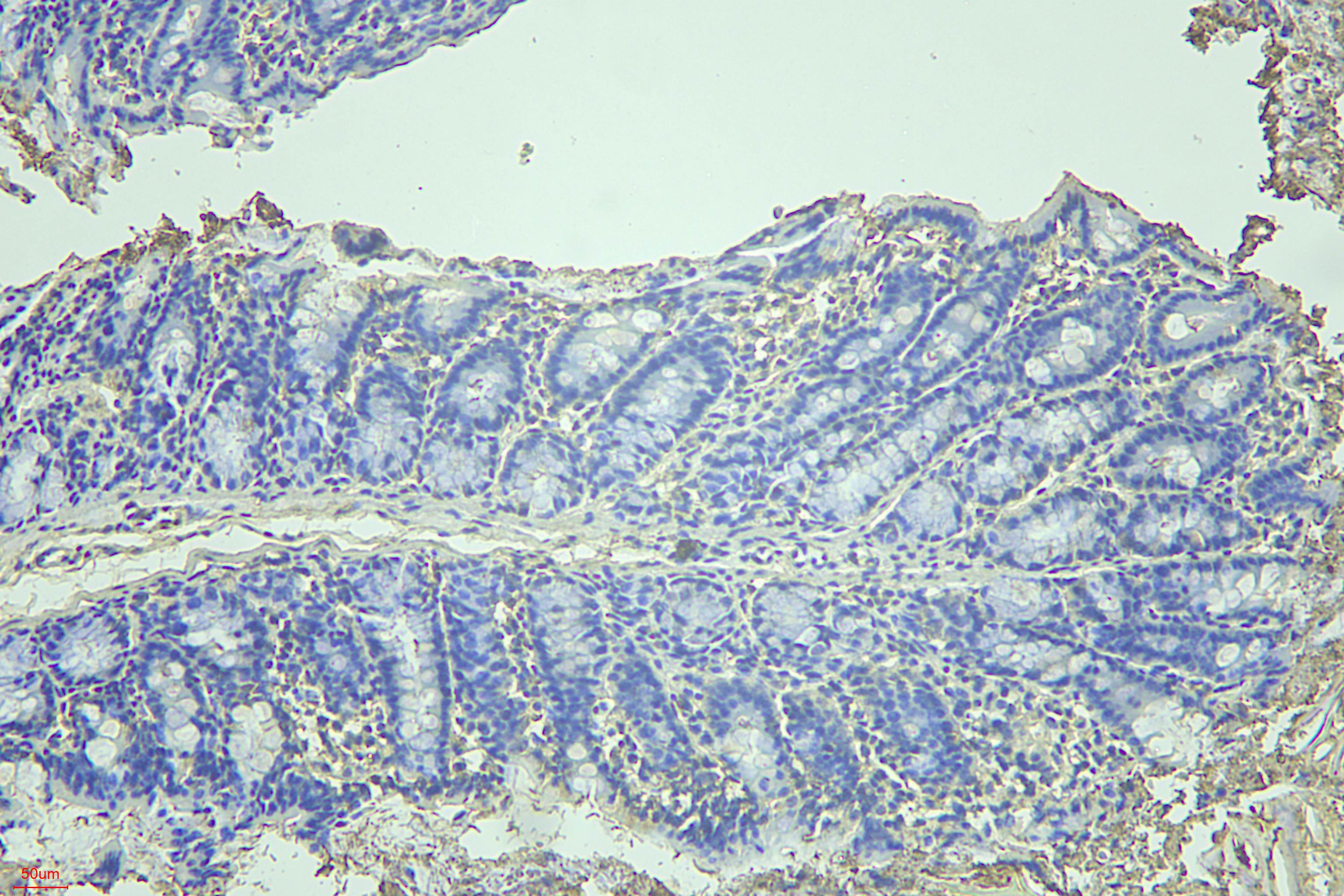

Supplement: Supplementary file 1 [file Data_Sheet_1.ZIP › raw data/Immunohistochemistry/Occludin/OA Occludin 100.jpg]

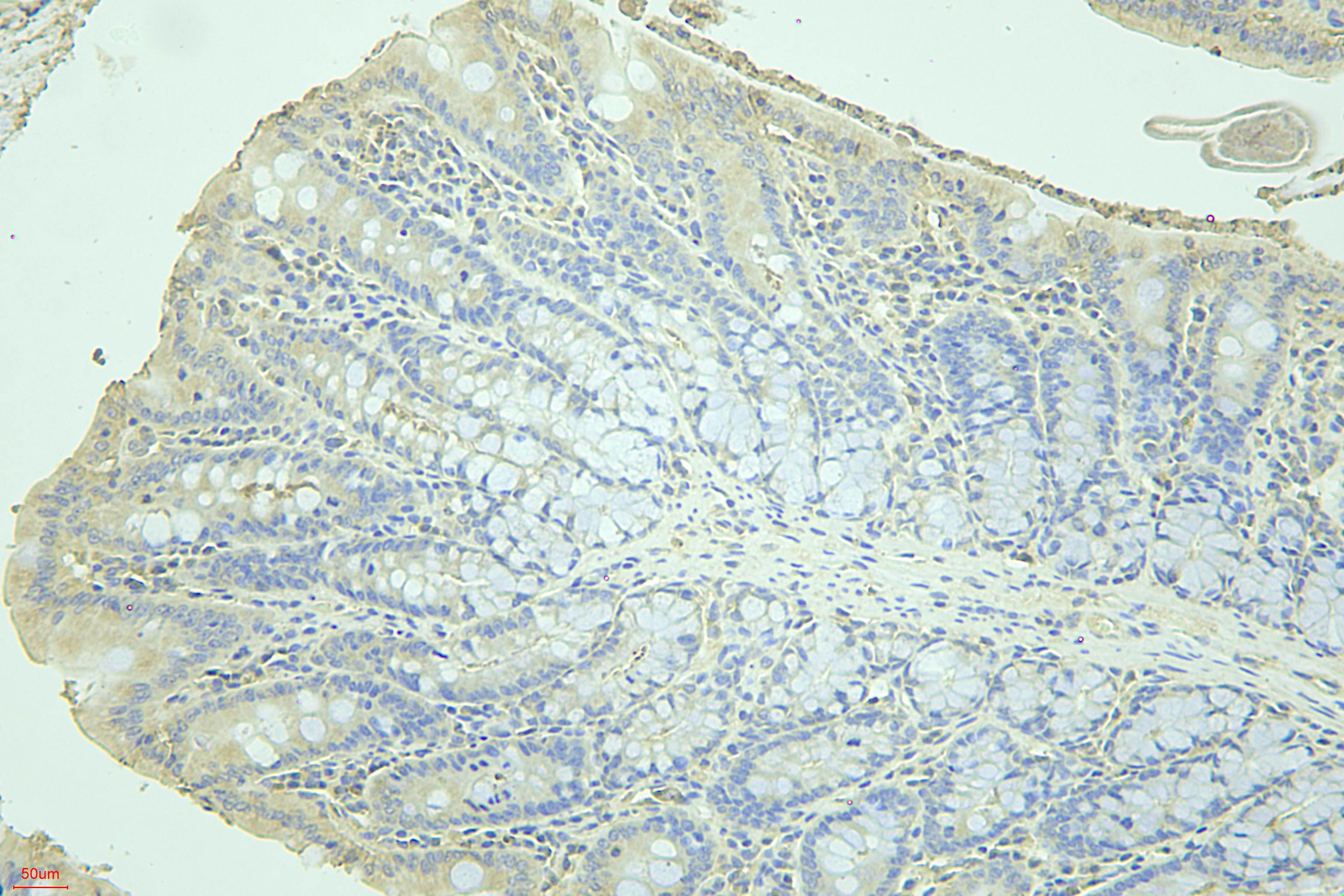

Supplement: Supplementary file 1 [file Data_Sheet_1.ZIP › raw data/Immunohistochemistry/ZO-1/TREAT ZO-1 100.jpg]

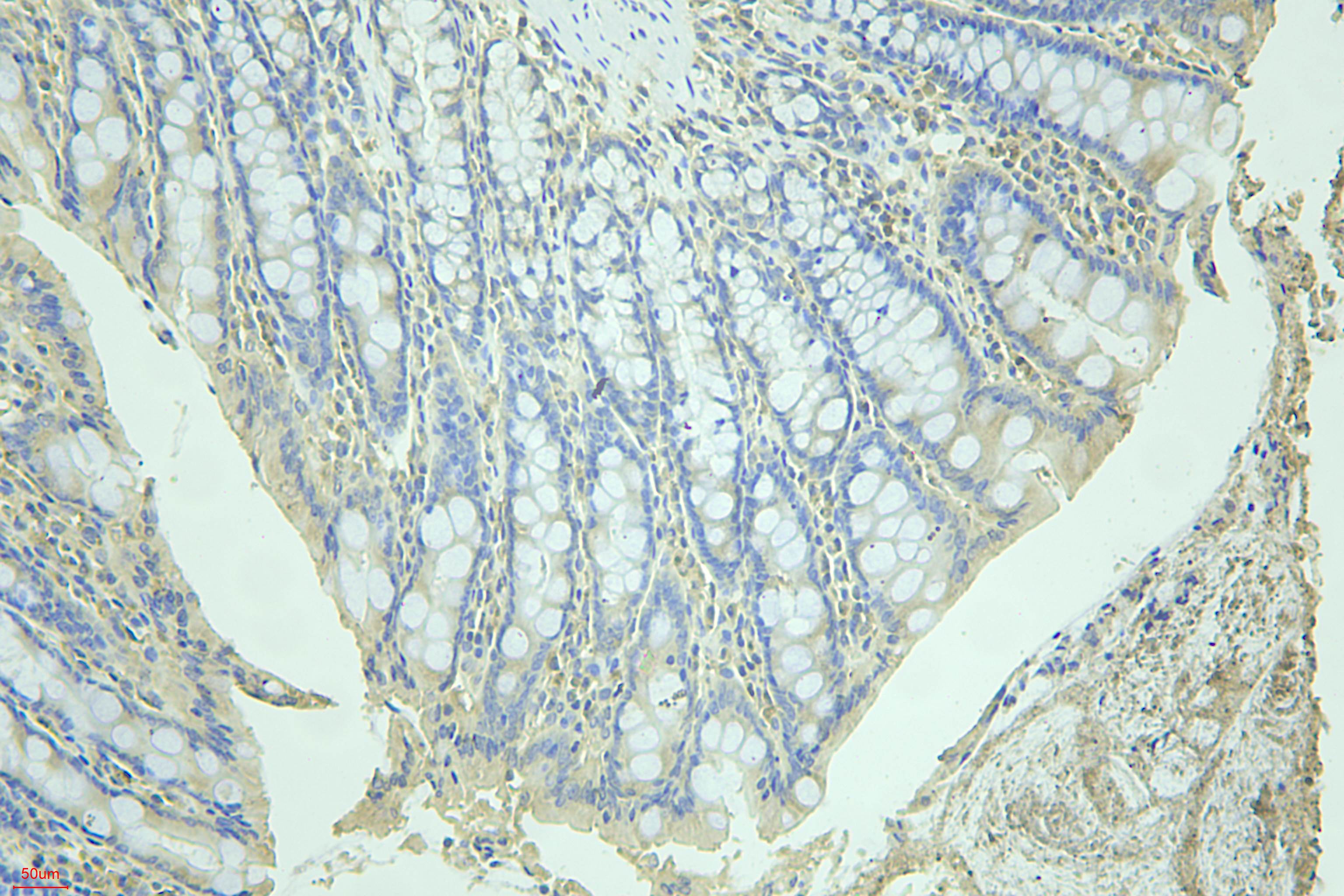

Supplement: Supplementary file 1 [file Data_Sheet_1.ZIP › raw data/Immunohistochemistry/ZO-1/CON ZO-1 100.jpg]

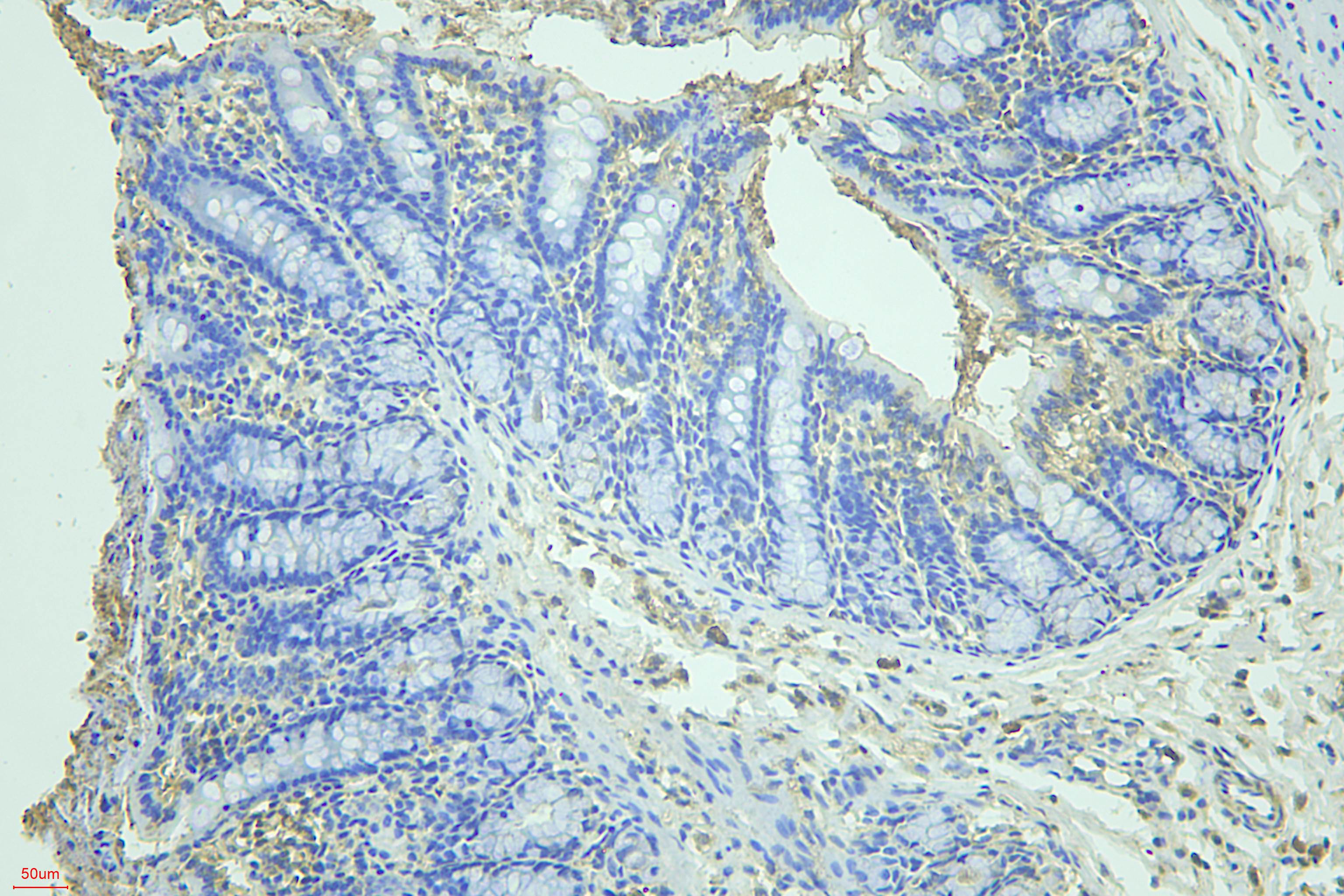

Supplement: Supplementary file 1 [file Data_Sheet_1.ZIP › raw data/Immunohistochemistry/ZO-1/OA ZO-1 100.jpg]
